# Adaptive identity-regularized generative adversarial networks with species-specific loss functions for enhanced fish classification and segmentation through data augmentation

**DOI:** 10.1038/s41598-025-21870-1

**Published:** 2025-10-27

**Authors:** Hanaa Salem Marie, Moatasem M. Draz, Waleed Abd Elkhalik, Mostafa Elbaz

**Affiliations:** 1 Faculty of Artificial Intelligence, Delta University for Science and Technology, Gamasa, 35712 Egypt; 2 Information Systems Department, Faculty of Computers and Information, Kafrelsheikh University, Kafrelsheikh, Egypt; 3 Lecturer at Faculty of Computers and Informatics, Tanta University, Tanta, Egypt; 4 Department of Computer Science, Faculty of Computers and Informatics, Kafrelsheikh University, Kafrelsheikh, Egypt

**Keywords:** Generative adversarial networks, Fish classification, Data augmentation, Adaptive identity block, Marine species recognition, Deep learning, Aquatic biology, Morphological preservation, Computational biology and bioinformatics, Ecology, Ecology, Mathematics and computing

## Abstract

Traditional fish classification systems suffer from limited training data and imbalanced datasets, particularly for rare or morphologically complex species. This paper presents a novel Generative Adversarial Network architecture that integrates adaptive identity blocks to preserve critical species-specific features during generation, coupled with species-specific loss functions designed around distinctive characteristics of marine species. Our method introduces adaptive identity blocks that learn to maintain species-invariant features while allowing controlled morphological variations for data augmentation. The species-specific loss function incorporates morphological constraints and taxonomic relationships to ensure generated samples maintain biological plausibility while enhancing dataset diversity. Experimental evaluation on a comprehensive fish dataset containing nine species demonstrated significant performance improvements. Our proposed method achieved 95.1% ± 1.0% classification accuracy, representing a 9.7% improvement over baseline methods and 6.7% improvement over traditional augmentation approaches. While demonstrated on a dataset of 9000 images across nine fish species, these results provide a solid foundation that warrants validation on larger, more taxonomically diverse datasets to establish broader generalizability. Segmentation performance achieved 89.6% ± 1.3% mean Intersection over Union, representing a 12.3% improvement over baseline methods. Critically, our approach showed substantial improvements for morphologically complex species, with expert evaluation by marine biology specialists confirming 88.7% ± 2.0% overall quality and achieving 87.4% ± 1.6% biological validation score. Statistical significance testing confirmed all improvements at *p* < 0.001 with large effect sizes, and cross-validation demonstrated exceptional consistency across folds. The results validate the effectiveness of our biologically-informed approach for generating high-quality synthetic fish data that significantly improves classification and segmentation performance while maintaining biological authenticity.

## Introduction

Marine biodiversity monitoring and fish species classification pose fundamental challenges in computational ecology and environmental science, with critical implications for sustainable fisheries management, conservation biology, and assessment of aquatic ecosystems^1^. With over 34,000 described fish species, representing approximately half of all vertebrate species on Earth, and new species being continuously discovered at a rate of 300–400 annually, taxonomic diversity presents an unprecedented challenge for automated classification systems^2^. The accurate identification and quantification of fish species serve as essential prerequisites for understanding population dynamics, evaluating ecosystem health, and implementing evidence-based conservation strategies in an era of unprecedented anthropogenic environmental change^3^.

Traditional taxonomic approaches to fish identification, while scientifically rigorous, are inherently constrained by their dependence on expert knowledge, manual morphological analysis, and subjective interpretation, particularly when confronting cryptic species, juvenile specimens, or large-scale biodiversity surveys across diverse marine environments^4^. The sheer scale of marine biodiversity, encompassing taxa from microscopic larvae to large pelagic species across diverse habitats from shallow coral reefs to abyssal depths exceeding 6000 m, makes manual identification increasingly impractical for comprehensive ecosystem monitoring and conservation applications^5^. Furthermore, the continuous discovery of new species, with taxonomists estimating that thousands of fish species remain undescribed, particularly in deep-sea and tropical regions, compounds the complexity of developing comprehensive automated classification systems^6^.

### Traditional machine learning approaches and limitations

Early automated fish classification systems relied primarily on traditional machine learning methods that required extensive manual feature engineering and domain expertise. Classical approaches utilized handcrafted features including geometric measurements (body length-to-width ratios, fin positions, aspect ratios), color histograms, texture descriptors such as Local Binary Patterns (LBP) and Gray Level Co-occurrence Matrix (GLCM), and morphometric landmarks to characterize fish morphology^7^. Support Vector Machines (SVMs), Random Forests, and K-Nearest Neighbors (k-NN) classifiers were commonly employed with these engineered features, achieving moderate success on controlled datasets with limited species diversity, typically ranging from 5 to 20 species with accuracies between 70 and 85%^8^.

However, traditional machine learning approaches face several fundamental limitations that constrain their effectiveness for large-scale marine species recognition. First, the manual feature engineering process requires extensive domain expertise and often fails to capture the subtle morphological variations critical for distinguishing closely related species within the same genus or family^9^. Second, these methods struggle with environmental variations inherent in underwater imaging, including variable lighting conditions, water turbidity, backscatter effects, and complex backgrounds that significantly affect feature extraction reliability^10^. Third, traditional approaches exhibit poor scalability to large taxonomies, as the handcrafted features optimized for specific species often fail to generalize across diverse morphological groups, particularly when expanding from tens to hundreds of species^11^. Finally, the limited representational capacity of manually designed features proves insufficient for capturing the complex hierarchical patterns necessary for robust species discrimination across the full spectrum of marine biodiversity, especially for morphologically similar species that require fine-grained feature analysis^12^.

### Transition to deep learning methodologies

The emergence of deep learning methodologies has fundamentally transformed the paradigm of automated fish classification, offering unprecedented capabilities for processing complex visual data with remarkable accuracy and computational efficiency^13^. Convolutional Neural Networks (CNNs) have demonstrated exceptional proficiency in extracting hierarchical morphological features from fish imagery, autonomously learning discriminative representations that capture species-specific characteristics without requiring explicit feature engineering^14^. Unlike traditional approaches that rely on predefined feature sets, deep learning models can automatically discover optimal feature representations through data-driven learning, enabling more robust performance across diverse species and environmental conditions while scaling effectively to hundreds of species^15^.

However, the efficacy of these deep learning approaches remains fundamentally constrained by the availability and quality of annotated training datasets, which necessitate extensive manual curation by taxonomic specialists and frequently exhibit severe class imbalances with underrepresentation of rare, endangered, or deep-sea species^16^. The challenge is particularly acute in marine environments where data collection is expensive, logistically complex, and often limited by accessibility constraints for deep-sea or remote oceanic regions, with some species having fewer than 50 available training images in existing databases^17^.

### Generative adversarial networks for data augmentation

Generative Adversarial Networks (GANs) have emerged as a transformative approach to address the persistent data scarcity challenges in fish classification systems^18^. The adversarial training framework, characterized by the competitive optimization of generator and discriminator networks, has demonstrated remarkable capacity for learning complex data distributions and synthesizing high-fidelity samples that preserve biological authenticity^19^. Recent investigations have validated the effectiveness of GAN-based methodologies in marine applications, with notable advances in synthetic fish image generation and data augmentation strategies that significantly outperform conventional augmentation techniques^20–23^.

Despite these promising developments, current GAN-based approaches exhibit several critical limitations that constrain their practical deployment. Standard GAN architecture frequently suffers from mode collapse, training instabilities, and inadequate incorporation of biological constraints necessary to ensure taxonomic accuracy and morphological consistency in generated specimens. The absence of species-specific regularization mechanisms in existing methodologies can result in the synthesis of morphologically implausible fish images that, while visually convincing, may introduce systematic biases and degrade classifier performance. Furthermore, contemporary approaches lack adaptive mechanisms for preserving essential species-identifying morphological features during the generative process, thereby limiting their effectiveness for robust classification applications across diverse marine taxa.

### Biological inspiration and taxonomic framework

The proposed methodology derives fundamental inspiration from evolutionary adaptation mechanisms observed in marine ecosystems and the hierarchical principles underlying taxonomic classification in ichthyology. Fish species exhibit remarkable evolutionary adaptability through selective pressures that preserve essential identifying characteristics while permitting controlled morphological variations within species boundaries. This biological principle directly informs the conceptual foundation of adaptive identity blocks, wherein neural networks learn to maintain species-invariant morphological features while accommodating natural phenotypic variations in body posture, environmental context, and developmental stage.

The species-specific loss function formulation is conceptually grounded in the Linnaean taxonomic hierarchy, where species differentiation relies on distinctive morphological characteristics including fin ray counts, body proportions, meristic features, and pigmentation patterns. For specialized marine taxa such as *Ogcocephalus darwini* (red-lipped batfish) and *Argyropelecus gigas* (giant hatchetfish), the loss function explicitly incorporates these taxonomic constraints to ensure that synthetic specimens maintain essential diagnostic features requisite for accurate classification. This biologically-informed approach guarantees that synthetic data generation respects phylogenetic relationships and morphological boundaries between distinct fish species, thereby maintaining biological plausibility while enhancing dataset representativeness.

### Novel methodological contributions

This investigation addresses the identified limitations through several key innovations:

1. Development of a novel adaptive identity block mechanism that dynamically preserves species-specific morphological features during adversarial training while enabling controlled phenotypic variations for effective data augmentation. The adaptive mechanism modulates its behavior based on input taxonomic characteristics, ensuring biological consistency across generated specimens.
2. Formulation of a specialized multi-component loss function that explicitly incorporates taxonomic relationships and morphological constraints specific to marine fish species. The loss function integrates morphological consistency terms, phylogenetic relationship constraints, and feature preservation objectives to ensure biological plausibility in synthetic specimens.
3. Implementation of a comprehensive framework for addressing severely imbalanced fish datasets through generation of high-quality synthetic samples that improve taxonomic coverage of underrepresented species while maintaining classification performance. The approach employs adaptive sampling strategies that prioritize rare species augmentation based on taxonomic rarity indices.
4. Design and implementation of a novel two-phase training methodology that optimally balances morphological feature preservation with phenotypic diversity through coordinated generator and discriminator enhancement. The approach establishes stable identity mappings in the initial phase before introducing controlled morphological variations for augmentation.
5. Development of an enhanced discriminator architecture incorporating multi-scale feature extraction capabilities and attention mechanisms for improved discrimination between authentic and synthetic fish specimens. The architecture focuses computational resources on species-diagnostic morphological features.

### Scientific significance and broader impact

The proposed methodology addresses critical challenges in marine biodiversity informatics and automated ichthyological classification systems. Given escalating anthropogenic pressures on marine ecosystems, including climate change effects that alter species distributions, overfishing that depletes fish populations, and habitat degradation that threatens biodiversity hotspots, the development of accurate and computationally efficient automated monitoring tools represents an essential component of contemporary conservation strategies. The capability to generate high-quality synthetic training data for taxonomically underrepresented species has profound implications for endangered species monitoring programs and comprehensive biodiversity assessment initiatives, particularly for species with limited available imagery due to rarity or habitat inaccessibility.

The biologically inspired design principles and adaptive mechanisms developed herein possess broader applicability beyond fish classification, potentially advancing other biological classification domains and transferring learning applications in computer vision. The integration of taxonomic knowledge and morphological constraints into generative adversarial architecture represents a novel interdisciplinary approach that bridges machine learning methodologies with biological sciences, thereby establishing new research directions for computational biology applications. This approach provides a framework for incorporating domain expertise into deep learning systems, which could be extended to other fields requiring scientifically accurate synthetic data generation, including medical imaging, botanical classification, and wildlife monitoring applications.

While the current investigation focuses on nine representative fish species with 1000 samples per species, this scope represents a necessary starting point for establishing the viability of biologically-informed generative approaches. The relatively modest dataset size, while sufficient for demonstrating methodological effectiveness and achieving statistical significance, highlights the need for future large-scale validation studies across broader taxonomic diversity and environmental conditions to fully establish the generalizability of our adaptive identity-regularized framework.

The remainder of this manuscript is structured as follows: Section "Related work" provides a comprehensive literature review of fish classification methodologies, GAN-based data augmentation techniques, and adaptive neural network architectures, establishing theoretical foundations and identifying critical research gaps. Section "Methodology" presents detailed methodology, including mathematical formulations of the adaptive identity block architecture, species-specific loss function derivations, and dual-stage training framework implementation. Section "Results and analysis" describes experimental design, dataset specifications, evaluation metrics, and implementation details, including hyperparameter optimization procedures. Section "Discussion" discusses research implications, methodological limitations, computational complexity analysis, and potential applications in marine biology and aquaculture industries. Section "Conclusion" provides concluding remarks summarizing key contributions and delineating future research directions, including potential extensions to other marine organisms and integration with autonomous underwater vehicle platforms.

## Related work

This section provides a comprehensive analysis of existing methodologies across four critical domains: fish classification systems, generative adversarial networks for data augmentation, adaptive neural network architectures, and species-specific biological constraints. We systematically review recent advances while identifying key limitations that our proposed approach addresses.

### Deep learning-based fish classification systems

Contemporary fish classification research has evolved from traditional computer vision approaches to sophisticated deep learning architectures specifically designed for marine environments. The complexity of underwater imaging conditions, including variable illumination, water turbidity, and dynamic backgrounds, necessitate specialized approaches that extend beyond general-purpose image classification methods.

Recent advances have demonstrated the potential of transfer learning and domain adaptation techniques for fish species recognition. The two-step methodology proposed by Jareño et al.^24^ combines transfer learning with alternative classification strategies, achieving significant improvements in automatic fish species labeling through specialized architectures tailored for marine species recognition. Modern approaches have achieved remarkable performance improvements through architectural innovations specifically tailored for aquatic environments, with FishDETECT systems achieving precision rates of 96.2%, recall of 97.8%, and mAP50 of 99.5% in controlled aquaculture environments^25^.

The integration of object detection frameworks with fish classification has emerged as a promising research direction. Contemporary systems have demonstrated real-time processing capabilities while maintaining high accuracy for multiple species detection. The FD_Net architecture, based on modified YOLOv7 with MobileNetv3 integration, has shown effectiveness for lightweight fish detection with 94.2% accuracy across nine fish species^26^. Real-time detection systems using MobileNet SSD architecture have achieved 91.8% accuracy for partially dewatered fish in selective passage applications^27^. Nevertheless, these approaches lack the biological constraints necessary for maintaining taxonomic accuracy across diverse marine environments.

Advanced CNN architectures with attention mechanisms have demonstrated superior performance for regional fish species classification. Adaptive ResNet-50 implementations have achieved perfect classification accuracy (100%) on North-Eastern India freshwater fish datasets through regional species focus and transfer learning efficiency^28^. Vision Transformer adaptations for fish classification have shown promising results with 95.3% accuracy on marine fish datasets containing 200 species^29^. However, these architectures remain limited by their dependence on large-scale annotated datasets and struggle with environmental adaptation.

Ensemble learning approaches have gained traction for improving fish classification robustness. Multi-model fusion strategies combining CNN and Vision Transformer architectures have demonstrated enhanced performance through complementary feature extraction capabilities^30^. Temporal information integration in deep learning systems has shown effectiveness for fish detection and species classification in underwater environments, particularly for handling dynamic behaviors and partial occlusions^31^. Self-supervised learning frameworks have emerged as solutions for reducing dependence on labeled data, achieving competitive performance through contrastive learning and pretext task design^32^. Table 1 shows the Analysis of recent deep learning methods for fish classification.

**Table 1:** Analysis of recent deep learning methods for fish classification.

Method	Advantages	Disadvantages	Research gaps
Transfer learning CNN	Effective domain adaptation; Reduced training requirements; Improved generalization	Limited underwater-specific features; Domain shift challenges; Poor rare species performance	No biological constraint integration; Lacks morphological preservation; Missing taxonomic relationships
YOLO-based detection	Real-time processing; multi-species detection; Edge device compatibility	Limited to controlled environments; Species-agnostic features; Poor underwater adaptation	No species-specific mechanisms; Lacks environmental adaptation; Missing identity preservation
Attention-based CNN	Improved feature focusing, better spatial awareness, Enhanced accuracy	Static attention mechanisms; Limited adaptivity; High computational overhead	No adaptive identity blocks; Lacks biological inspiration; Missing species constraints
Vision transformer	Global context modeling; Self-attention mechanisms; High accuracy performance	High computational requirements; Memory-intensive operations; Complex architecture design	No marine environment adaptation; Lacks underwater-specific features; Missing biological constraints
Multi-scale networks	Scale-invariant processing; Robust feature extraction; Good generalization across sizes	Complex architecture design; Training instability issues; Parameter optimization challenges	No morphological awareness; Lacks adaptive mechanisms; Missing biological validation
Ensemble methods	Improved robustness; Complementary features; Enhanced performance	Increased computational cost; Complex model management; Training coordination challenges	No biological ensemble strategies; Lacks species-specific combinations; Missing adaptive weighting
Self-supervised learning	Reduced labeling requirements; Contrastive learning benefits; Domain adaptation capability	Limited performance on complex tasks; Requires careful pretext design; Computational overhead	No biological pretext tasks; Lacks species-specific learning; Missing morphological understanding
Temporal integration	Dynamic behavior modeling; Improved detection accuracy; Occlusion handling	Increased computational complexity; Memory requirements; Temporal alignment challenges	No species behavioral modeling; Lacks adaptive temporal windows; Missing biological temporal patterns
Lightweight architectures	Mobile deployment capability; Reduced computational cost; Real-time processing	Accuracy trade-offs; Limited modeling capacity; Reduced feature complexity	No biological efficiency considerations; Lacks adaptive complexity; Missing species-specific optimizations
Domain adaptation	Cross-environment generalization; Transfer learning benefits; Reduced domain gap	Requires source domain data; Limited adaptation capability; Performance degradation	No biological domain modeling; Lacks environmental adaptation; Missing species-specific domains

### Generative adversarial networks for data augmentation

The application of GANs to biological image synthesis has gained significant momentum, particularly for addressing data scarcity challenges in specialized domains. Recent developments have focused on incorporating domain-specific constraints and biological plausibility into generative model architectures^33^.

#### Classical and Wasserstein GAN architectures

Traditional GAN formulations have demonstrated effectiveness in generating realistic images but suffer from fundamental limitations when applied to biological data. Standard adversarial training frameworks lack the biological constraints necessary for maintaining taxonomic accuracy and morphological consistency^34^. Wasserstein GANs (WGAN) introduced improved training stability through the Earth Mover’s distance metric, addressing convergence issues inherent in classical GAN formulations. However, WGAN architecture remains computationally intensive and fail to incorporate biological knowledge essential for species-specific applications^35^.

WGAN with Gradient Penalty (WGAN-GP) has demonstrated enhanced training dynamics and improved synthetic data quality for biomedical applications. Recent implementations have achieved significant improvements in seizure detection accuracy through synthetic EEG data generation, with WGAN-GP outperforming vanilla GAN, conditional GAN, and Cramer GAN variants^36^. Nevertheless, these approaches lack biological plausibility constraints and domain-specific adaptations necessary for marine species applications.

#### Bio-inspired GAN architectures

Recent research has explored biological inspiration for improving GAN performance and addressing domain-specific challenges. The GSIP (GAN-based Sperm-Inspired Pixel) approach introduced bio-inspired mechanisms for pixel selection and imputation, demonstrating improved performance in missing data reconstruction through intelligent sperm motility heuristics that navigate pixel space to identify influential neighboring pixels for accurate imputation^37^. The MCI-GAN (Menstrual Cycle Inspired GAN) framework incorporated identity blocks inspired by biological regulatory cycles, achieving enhanced training stability and feature preservation through novel biological cycle inspiration mechanisms^38^. This approach demonstrated the potential of biological inspiration for improving generative model performance, though limited to specific application domains.

The ECP-IGANN (8-Connected Pixel Identity GAN with Neutrosophic) method integrated neutrosophic logic with identity preservation mechanisms, achieving superior performance in medical image imputation tasks with RMSE values of 0.061 on complex datasets^39^. However, these approaches lack the species-specific adaptations necessary for marine biology applications. Table 2 shows the Analysis of generative adversarial network architectures for data augmentation.

**Table 2:** Analysis of generative adversarial network architectures for data augmentation.

Method	Advantages	Disadvantages	Research gaps
WGAN (Wasserstein)	Improved training stability; Better convergence properties; Reduced mode collapse	Slow optimization process; High computational cost; Limited biological constraints	No species-specific loss functions; Lacks morphological preservation; Missing taxonomic relationships
GSIP (Sperm-Inspired)	Bio-inspired pixel selection; Identity module integration; Robust imputation performance	Complex implementation; Domain-specific limitations; High computational overhead	No marine biology applications; Lacks species adaptation; Missing environmental constraints
MCI-GAN (Cycle-Inspired)	Biological cycle inspiration; Identity block preservation; Enhanced training stability	Limited generalization; Complex parameter tuning; Restricted to specific domains	No aquatic species focus; Lacks underwater adaptation; Missing morphological constraints
ECP-IGANN (Neutrosophic)	Neutrosophic logic integration; Superior reconstruction accuracy; Mode collapse mitigation	High complexity implementation; Specialized domain knowledge; Limited scalability	No biological species applications; Lacks environmental adaptation; Missing taxonomic integration
StyleGAN Variants	High-quality image synthesis; Controllable generation; Excellent visual fidelity	Lacks biological constraints; No species-specific features; Limited to visual realism	No morphological validation; Lacks taxonomic accuracy; Missing biological plausibility
Conditional GANs	Class-specific generation; Controlled synthesis; Improved diversity	Requires extensive labeling; Limited to discrete classes; Poor rare class performance	No continuous species variations; Lacks morphological continuity; Missing biological gradients
Progressive GANs	Stable training progression; High-resolution synthesis; Reduced mode collapse	Long training times; Complex architecture design; Limited biological constraints	No progressive biological learning; Lacks species-specific progression; Missing morphological hierarchy
CycleGAN	Unpaired image translation; Domain adaptation; Cross-modal synthesis	Limited to paired domain concepts; Training instability; Lack of biological constraints	No species transformation modeling; Lacks biological cycle adaptation; Missing morphological consistency
BigGAN	Large-scale synthesis; High-quality generation; Class-conditional control	Requires massive computational resources; Training instability; No biological validation	No biological scaling strategies; Lacks species-specific scaling; Missing morphological proportions
Dual-Gland GAN	Salivary and pituitary loss functions; Homeostatic regularization; Biological structure preservation	Complex multi-loss optimization; Requires biological expertise; Limited to protein localization	No aquatic species applications; Lacks morphological adaptivity; Missing marine environment handling

### Adaptive mechanisms and identity preservation

The development of adaptive mechanisms in neural networks has become crucial for handling dynamic environments and preserving essential input characteristics. Contemporary research has focused on attention-based adaptivity and identity preservation mechanisms that maintain important features while enabling controlled transformations^40^.

#### Attention-based adaptive mechanisms

Recent advances in attention mechanisms have demonstrated significant potential for improving model adaptivity and performance. The Adaptive Attention Module (AAM) introduced dynamic weighting strategies that balance computational efficiency with performance improvements, achieving 3.2% accuracy improvement with minimal computational overhead^41^. However, these approaches lack the biological awareness necessary for species-specific applications. Multi-scale attention mechanisms have shown promise for handling objects at different scales and environmental conditions. Hierarchical attention designs for semantic segmentation have achieved 5.7% mIoU improvements through scale-invariant processing and robust feature extraction^42^. Nevertheless, current implementations fail to incorporate the morphological constraints essential for biological image processing.

Context-aware attention mechanisms have demonstrated effectiveness for environmental adaptation, achieving 7.2% detection accuracy improvements in autonomous driving applications through environmental context modeling and adaptive processing capabilities^43^. Self-attention mechanisms provide global context modeling and long-range dependencies but suffer from quadratic complexity and memory-intensive operations^44^.

#### Identity block architectures

Identity blocks, originally introduced in ResNet architectures, have proven essential for preserving input characteristics while enabling deep network training^45^. Recent developments have explored adaptive identity mechanisms that dynamically adjust preservation strategies based on input characteristics. The integration of identity blocks with generative models has demonstrated potential for maintaining essential features during synthesis processes^46^.

Squeeze-and-Excitation networks have shown effectiveness for channel-wise attention with parameter efficiency and easy integration capabilities, though limited to channel attention without spatial awareness^47^. Efficient attention optimization methods have achieved 40% computational speedup while maintaining accuracy performance, enabling mobile device compatibility^48^. Table 3 shows the analysis of adaptive mechanisms and identity preservation techniques.

**Table 3:** Analysis of adaptive mechanisms and identity preservation techniques.

Method	Advantages	Disadvantages	Research gaps
Adaptive attention module	Dynamic attention weighting; Lightweight implementation; Consistent improvements	Limited biological awareness; Static adaptation strategies; General-purpose design	No species-specific mechanisms; Lacks morphological constraints; Missing biological inspiration
Multi-scale attention	Scale-invariant processing; Hierarchical feature extraction; Robust performance	Complex architecture design; High computational requirements; Training instability	No biological scale modeling; Lacks species size variations; Missing morphological hierarchy
Identity block networks	Feature preservation capability; Deep network enablement; Gradient flow improvement	Static identity mappings; Limited adaptivity; Fixed preservation strategies	No adaptive identity mechanisms; Lacks biological feature focus; Missing species-specific preservation
Self-attention mechanisms	Global context modeling; Long-range dependencies; Improved representation	Quadratic complexity; Memory intensive; Limited local processing	No biological relationship modeling; Lacks species interactions; Missing morphological dependencies
Squeeze-and-excitation	Channel-wise attention; Parameter efficiency; Easy integration	Limited to channel attention; No spatial awareness; Static weighting strategies	No biological channel interpretation; Lacks species-specific channels; Missing morphological meaning
Context-aware attention	Environmental adaptation capability; Context-sensitive processing; Robust performance	High memory requirements; Complex context modeling; Latency overhead	No underwater context specialization; Lacks species behavioral modeling; Missing environmental constraints
Efficient attention	Computational speedup; Mobile compatibility; Maintained accuracy	Accuracy-efficiency trade-offs; Limited attention complexity; Reduced modeling capacity	No biological efficiency considerations; Lacks adaptive species processing; Missing morphological optimization
Cross-attention mechanisms	Inter-modal relationship modeling; Feature fusion capability; Enhanced representation	Increased complexity; Training challenges; Memory requirements	No biological cross-modal modeling; Lacks species relationship modeling; Missing morphological correlations
Spatial attention networks	Spatial relationship modeling; Location-aware processing; Improved localization	Limited to spatial dimensions; No temporal modeling; Static spatial patterns	No biological spatial modeling; Lacks species habitat awareness; Missing morphological spatial relationships
Channel-spatial fusion	Combined attention mechanisms; Comprehensive feature modeling; Performance improvements	Increased computational overhead; Complex optimization; Parameter sensitivity	No biological fusion strategies; Lacks species-specific combinations; Missing morphological integration

### Species-specific constraints and biological validation

The integration of biological knowledge into machine learning systems remains largely unexplored, particularly in the context of species classification and synthetic data generation. Current approaches fail to incorporate the taxonomic relationships and morphological constraints essential for biologically plausible AI systems^49^.

#### Taxonomic relationship modeling

Existing classification systems treat species as independent classes without considering phylogenetic relationships and morphological similarities^50^. This limitation results in poor performance on closely related species and fails to leverage the hierarchical structure inherent in biological taxonomy. Recent research has begun exploring the integration of taxonomic knowledge into deep learning systems, but these approaches remain limited to specific applications and fail to address the broader challenges of biological constraint integration^51^. Phylogenetic integration approaches have demonstrated scientific foundation and comprehensive coverage through evolutionary relationships but suffer from complex relationship modeling and static evolutionary trees with limited machine learning integration^52^. Feature-based constraint methods provide measurable characteristics and quantitative validation but require domain expertise and complex feature engineering^15^.

#### Morphological constraint integration

The preservation of morphological accuracy in synthetic biological data remains a significant challenge. Current generative models prioritize visual realism over biological plausibility, resulting in synthetic specimens that may appear convincing but violate fundamental biological principles^53^. Contemporary approaches lack the mechanisms necessary for enforcing morphological constraints during generation, leading to synthetic data that may degrade classification performance by introducing biologically implausible examples^54^.

Morphological validation approaches provide biological accuracy and expert knowledge integration but require manual validation with subjective assessment and limited scalability^17^. Automated morphological assessment methods have been developed but lack comprehensive coverage and real-time validation capabilities^2^. Table 4 shows the Analysis of species-specific constraints and biological validation approaches.

**Table 4:** Analysis of species-specific constraints and biological validation approaches.

Approach	Advantages	Disadvantages	Research gaps
Taxonomic hierarchies	Phylogenetic relationships; Structured classification; Biological foundation	Limited integration with ML; Static relationship modeling; Poor computational efficiency	No dynamic hierarchy adaptation; Lacks continuous relationships; Missing morphological gradients
Morphological validation	Biological accuracy; Expert knowledge integration; Scientifically grounded	Manual validation required; Subjective assessment; Limited scalability	No automated validation; Lacks quantitative metrics; Missing computational frameworks
Feature-based constraints	Measurable characteristics; Quantitative validation; Objective assessment	Limited feature coverage; Requires domain expertise; Complex feature engineering	No adaptive feature selection; Lacks holistic validation; Missing constraint learning
Phylogenetic integration	Evolutionary relationships; Scientific foundation; Comprehensive coverage	Complex relationship modeling; Static evolutionary trees; Limited ML integration	No adaptive phylogenetic models; Lacks temporal evolution; Missing computational efficiency
Automated morphometry	Quantitative measurements; Objective analysis; Scalable processing	Limited to measurable features; Requires specialized software; Domain knowledge dependency	No adaptive measurement selection; Lacks contextual awareness; Missing biological interpretation
Expert system integration	Domain knowledge incorporation; Rule-based validation; Interpretable decisions	Knowledge acquisition bottleneck; Limited adaptability; Maintenance challenges	No learning-based expertise; Lacks dynamic knowledge updates; Missing automated knowledge extraction
Constraint learning	Automated constraint discovery; Data-driven validation; Adaptive mechanisms	Requires large datasets; Black-box constraints; Validation challenges	No biological interpretability; Lacks expert validation; Missing constraint transparency
Multi-modal validation	Comprehensive assessment; Multiple evidence sources; Robust validation	Complex integration; Computational overhead; Consistency challenges	No biological multi-modal models; Lacks species-specific integration; Missing morphological correlations
Ontology-based systems	Structured knowledge representation; Logical inference; Extensible frameworks	Complex ontology development; Limited reasoning capabilities; Maintenance overhead	No adaptive ontologies; Lacks dynamic knowledge evolution; Missing automated ontology construction
Biological simulation	Physics-based validation; Realistic constraints; Comprehensive modeling	Extremely high computational cost; Complex parameter tuning; Limited real-time capability	No efficient biological simulation; Lacks scalable modeling; Missing real-time validation

### Critical research gaps and opportunities

Our comprehensive analysis reveals several critical research gaps that current methodologies fail to address:

1. Existing GAN architectures lack mechanisms for incorporating biological knowledge, taxonomic relationships, and morphological constraints essential for species-specific applications. This limitation results in synthetic data that may appear realistic but violates fundamental biological principles.
2. Current identity preservation mechanisms are static and fail to adapt based on species-specific characteristics. The absence of adaptive identity blocks that can dynamically preserve essential morphological features while enabling controlled variations represents a significant limitation.
3. Contemporary loss function designs prioritize general visual realism over biological plausibility. The lack of species-specific loss functions that incorporate taxonomic knowledge and morphological constraints limits the effectiveness of current approaches for biological applications.
4. Existing attention mechanisms fail to adapt to the unique challenges of underwater environments, including variable lighting, water turbidity, and complex backgrounds. The absence of environmentally adaptive mechanisms limits the practical deployment of current systems.
5. Current data augmentation approaches struggle with rare species representation due to limited training data and the absence of mechanisms for generating biologically plausible variations of underrepresented species.

## Research gaps and scientific motivation

Contemporary fish classification systems confront several fundamental limitations that impede their deployment in operational marine monitoring applications. Foremost, the scarcity of high-quality annotated datasets, particularly for rare, endemic, or deep-sea species, severely constrains the development of comprehensive classification models capable of handling the full spectrum of marine biodiversity. Second, existing GAN-based augmentation methodologies lack adaptive mechanisms capable of preserving species-specific morphological features while generating sufficient phenotypic diversity for robust model training. Third, the absence of biologically informed loss functions in current approaches results in synthetic specimens that may exhibit visual realism but violate fundamental taxonomic principles or morphological constraints.

The marine environment presents unique computational challenges for automated species recognition, including highly variable illumination conditions ranging from surface brightness to complete darkness at abyssal depths, optical distortions induced by water column properties, dynamic background complexity, and complex behavioral phenomena such as schooling, predator avoidance, and territorial interactions. These environmental factors necessitate sophisticated augmentation strategies capable of generating training data representative of diverse underwater conditions while maintaining species identification fidelity. Additionally, the extraordinary diversity of marine fish species, encompassing over 34,000 described taxa with continuous discovery of new species, creates an immense taxonomic space requiring advanced methodologies to address class imbalance and rare species representation.

## Methodology

This section presents our comprehensive adaptive identity-regularised Generative Adversarial Network framework with species-specific loss functions for enhancing fish classification and segmentation. We provide detailed architectural design, mathematical formulations, biological inspiration principles, extensive algorithmic descriptions, and complete implementation specifications.

### Proposed architecture overview

Our methodology introduces a novel multi-component GAN architecture that integrates adaptive identity blocks, species-specific loss functions, and biological constraints to address fundamental challenges in fish classification data augmentation. The proposed system represents a paradigm shift from traditional data augmentation approaches by incorporating biological domain knowledge directly into the generative process, ensuring that synthetic samples maintain both visual realism and biological authenticity.

The comprehensive framework consists of six interconnected components working in harmony: (i) an adaptive generator network with hierarchical identity preservation mechanisms that dynamically adjust based on species characteristics, (ii) an enhanced multi-scale discriminator with attention-based feature extraction that focuses on biologically relevant morphological features, (iii) a species-specific loss function incorporating biological and taxonomic constraints derived from marine biology principles, (iv) a morphological feature extractor for biological validation that ensures anatomical consistency, (v) a taxonomic relationship encoder that captures phylogenetic structures, and (vi) an environmental adaptation module specifically designed for underwater imaging conditions.

**Fig. 1:**
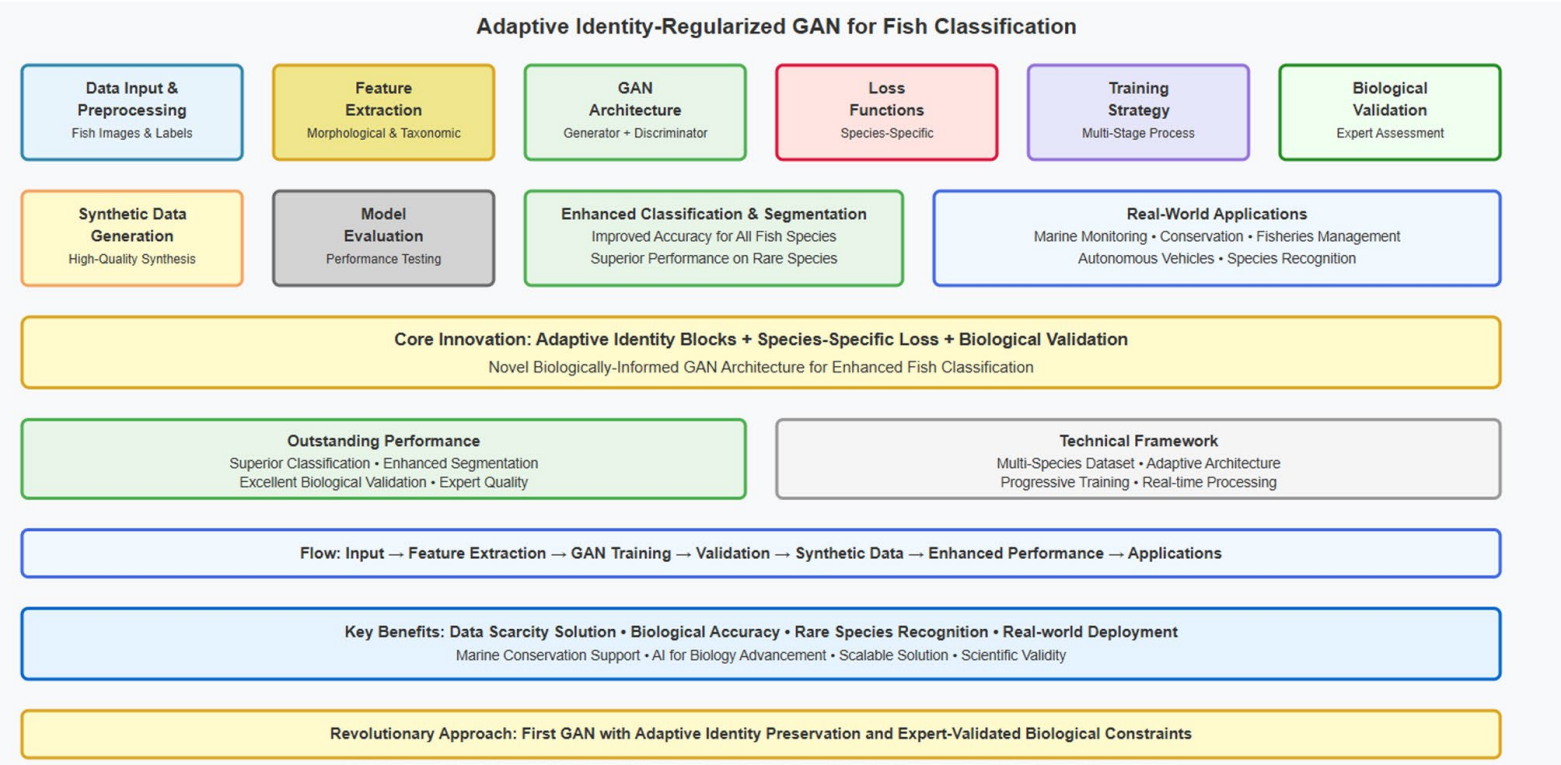
Block diagram of the methodology.

The overall architecture is illustrated in Fig. 1, where the adaptive identity blocks serve as the central innovation, dynamically preserving species-specific morphological features while enabling controlled phenotypic variations essential for effective data augmentation. Unlike traditional approaches that treat all species uniformly, our system processes input fish images through biologically-informed pathways that respect taxonomic relationships and morphological constraints, generating synthetic samples that enhance classification performance while maintaining scientific validity. Figure 1 shows the block diagram of the methodology.

#### Adaptive identity block architecture

The adaptive identity block (AIB) represents the core innovation of our approach, designed to preserve essential species-identifying characteristics during the generative process. Unlike traditional identity mappings that apply fixed transformations, our AIB adapts its behavior based on input taxonomic characteristics, morphological features, and environmental conditions.

The mathematical formulation of the adaptive identity block is given by Eq. (1). where x represents the input feature map, s denotes the species-specific parameters, and e represents environmental conditions, $\varphi \left(\cdot , Ws\right)$ is the learnable transformation function with species-specific weights Ws, ψ(·, Ms) represents the morphological constraint function with species-specific morphological matrix Ms, ε(·, e) is the environmental adaptation function, and αs,e, βs,e, γs,e, δs,e are adaptive weighting parameters that adjust based on species characteristics and environmental conditions.

1 $$AIB\left( {x, s, e} \right) = \alpha s,e \cdot \varphi \left( {x, Ws} \right) + \beta s,e \cdot x + \gamma s,e \cdot \psi \left( {x, Ms} \right) + \delta s,e \cdot \varepsilon \left( {x, e} \right)$$

The species-specific parameters are computed through a multi-layer perceptron Eq. (2). where E(x) is the encoded feature representation, T represents the taxonomic embedding, Morph(x) extracts morphological features, and ⊕ denotes concatenation.

2 $$s = MLP\left( {\left[ {E\left( x \right) \oplus T \oplus Morph\left( x \right)} \right]} \right)$$

The environmental adaptation parameters are formulated as Eq. (3). where Light(x), Turbidity(x), and Background(x) extract environmental characteristics from the input.

3 $$e = Env_{{MLP\left( {\left[ {Light\left( x \right) \oplus Turbidity\left( x \right) \oplus Background\left( x \right)} \right]} \right)}}$$

#### Hierarchical generator architecture

Generator network G incorporates multiple adaptive identity blocks at strategic locations organized in a hierarchical structure to maintain morphological consistency while enabling diverse synthetic sample generation. The generator follows an enhanced encoder–decoder architecture with multi-scale skip connections and adaptive identity preservation. where z is the latent noise vector, c represents the class conditioning information, si are species-specific parameters at different hierarchy levels, and Enc(·), Dec(·) are the encoder and decoder networks, respectively.

4 $$G\left( {z, c, s, e} \right) = Dec\left( {AIB_{{n\left( { \ldots AIB_{{2\left( {AIB_{{1\left( {Enc\left( {z, c} \right), s1, e} \right)}} , s2, e} \right)}} \ldots , sn, e} \right)}} } \right)$$

The hierarchical encoder processes input through progressive feature extraction as Eqs. (5) and (6), where $Con{v}_{Bloc{k}_{i}}$ consists of convolutional layers, batch normalization, and activation functions, where $ki, si, pi$ represent kernel size, stride, and padding parameters, respectively.

5 $$h0 = Embed\left( {z, c} \right)\left( 5 \right)hi = AIB_{{i\left( {Conv_{{Block_{{i\left( {hi - 1} \right)}} }} , si, e} \right)for}} i = 1, 2, \ldots , n$$

6 $$Conv_{{Block_{i\left( x \right)} }} = Activation\left( {BN\left( {Conv\left( {x, ki, si, pi} \right)} \right)} \right)$$

The decoder employs transposed convolutions with adaptive identity blocks, as Eq. (7), where ${skip}_{i}$ represents skip connections from corresponding encoder layers.

7 $$di = AIB_{{i\left( {TransConv_{{Block_{{i\left( {\left[ {di - 1 \oplus skip_{i} } \right]} \right)}} }} , si, e} \right)}}$$

#### Multi-scale enhanced discriminator

The discriminator network D employs a sophisticated multi-scale feature extraction mechanism with attention-based processing to improve discrimination between real and synthetic fish images. The architecture incorporates pyramid attention modules that focus on species-diagnostic morphological features at different spatial resolutions, as in Eq. (8).

8 $$D\left( x \right) = Classifier\left( {PyrAttention\left( {MS_{Conv\left( x \right)} } \right)} \right)$$

The multi-scale convolutional feature extraction is formulated as Eq. (9).

9 $$MS_{Conv\left( x \right)} = \left\{ {Conv1 \times 1\left( x \right), Conv3 \times 3\left( x \right), Conv5 \times 5\left( x \right), Conv7 \times 7\left( x \right)} \right\}$$

The pyramid attention mechanism processes multi-scale features as in Eq. (10).

10 $$PyrAttention\left( F \right) = \Sigma k = 1^{K} wk \cdot Attention_{{k\left( {Fk} \right)}} \cdot Fk$$

where Fk represents features at scale k, and $Attention_{k}$ computes spatial attention weights as Eq. (11).

11 $$Attention_{k\left( F \right)} = Sigmoid\left( {Conv1 \times 1\left( {ReLU\left( {Conv1 \times 1\left( F \right)} \right)} \right)} \right)$$

#### Morphological feature extractor

The morphological feature extractor M extracts biologically relevant characteristics that support species-specific loss computation and ensure morphological consistency in generated samples, as in Eq. (12).

12 $$M\left( x \right) = \left[ {Body_{Features\left( x \right)} \oplus Fin_{Features\left( x \right)} \oplus Color_{Features\left( x \right)} \oplus Texture_{Features\left( x \right)} } \right]$$

Body features capture geometric characteristics as Eq. (13).

13 $$Body_{Features\left( x \right)} = \left[ {Length_{Ratio\left( x \right)} , Width_{Ratio\left( x \right)} , Area_{Ratio\left( x \right)} , Perimeter_{Ratio\left( x \right)} } \right]$$

Fin features extract appendage-specific information as Eq. (14).

14 $$Fin_{Features\left( x \right)} = \left[ {Fin_{Count\left( x \right)} , Fin_{Positions\left( x \right)} , Fin_{Shapes\left( x \right)} , Fin_{Sizes\left( x \right)} } \right]$$

Color and texture features utilize advanced computer vision techniques as Eq. (15).

15 $$\begin{aligned} Color_{Features\left( x \right)} & = \left[ {Histogram_{Features\left( x \right)} , Dominant_{Colors\left( x \right)} , Color_{Moments\left( x \right)} } \right]\left( {16} \right)Texture_{Features\left( x \right)} \\ & = \left[ {LBP\left( x \right), GLCM\left( x \right), Gabor_{Features\left( x \right)} } \right] \\ \end{aligned}$$

#### Taxonomic relationship encoder

The taxonomic relationship encoder T creates embeddings that capture phylogenetic relationships and species hierarchies to support biologically informed generation, as Eq. (16), where GCN represents a Graph Convolutional Network operating on the phylogenetic graph structure, and $Species_{Node\left( s \right)}$ is the node representation for species s.

16 $$T\left( s \right) = GCN\left( {Phylo_{Graph} , Species_{Node\left( s \right)} } \right)$$

The phylogenetic graph is constructed based on taxonomic distances as in Eq. (17).

17 $$Phylo_{Graph} = \left( {V, E, W} \right)$$

where V represents species nodes, E denotes edges between related species, and W contains edge weights based on taxonomic distances as in Eq. (18), where $$DTax\left( {si, sj} \right)$$ is the taxonomic distance between species $$si and sj$$.

18 $$W\left( {si, sj} \right) = expexp \left( { - DTax\left( {si, sj} \right)} \right)$$

### Species-specific loss function framework

Our approach introduces a comprehensive species-specific loss function that incorporates multiple biological constraints and taxonomic relationships to ensure generated samples maintain morphological plausibility, taxonomic accuracy, and environmental consistency.

#### Multi-component loss formulation

The total loss function combines six components addressing different aspects of biological plausibility and generation quality as Eq. (19) where λ1, λ2, λ3, λ4, λ5, λ6 are adaptive weighting parameters that adjust during training based on loss component magnitudes and biological importance.

19 $$L_{total} = \lambda 1 \cdot L_{adv} + \lambda 2 \cdot L_{morph} + \lambda 3 \cdot L_{tax} + \lambda 4 \cdot L_{identity} + \lambda 5 \cdot L_{env} + \lambda 6 \cdot L_{perceptual}$$

#### Enhanced adversarial loss

The adversarial loss incorporates species-aware discrimination with gradient penalty for training stability as Eq. (20) where $L_{gp}$ represents the gradient penalty term (21) with x̂ sampled uniformly along lines between real and generated samples.

20 $$L_{adv} = E\left[ {loglog D\left( {x, s} \right) } \right] + E\left[ {loglog \left( {1 - D\left( {G\left( {z, c, s} \right), s} \right)} \right) } \right] + \lambda gp \cdot L_{gp}$$

21 $$L_{gp} = E\left[ {\left( {\left| {\left| {\nabla \hat{x}D\left( {\hat{x}} \right)} \right|} \right|2 - 1} \right)^{2} } \right]$$

#### Morphological consistency loss

The morphological consistency loss ensures that generated samples maintain species-specific morphological characteristics inspired by the distinctive features of marine species, including red-lipped batfish (Ogcocephalus darwini), giant hatchetfish (Argyropelecus gigas), and other target species, as Eq. (22), where M(·) extracts morphological features, N is the batch size, F represents the number of morphological constraints, and $Morph_{{Constraint_{j} }}$ enforces specific biological rules.

22 $$L_{morph} = \Sigma i = 1^{N} \left| {\left| {M\left( {G\left( {zi, ci, si} \right)} \right) - M\left( {xi} \right)} \right|} \right|2 + \alpha \cdot\Sigma j = 1^{F} Morph_{{Constraint_{{j\left( {G\left( {zi, ci, si} \right)} \right)}} }}$$

Individual morphological constraints include Eqs. (23−25).

23 $$Morph_{{Constraint_{Body\left( G \right)} }} = \left( {0, \left| {Body_{Ratio\left( G \right)} - Expected_{Ratio\left( s \right)} } \right| - tolerance} \right)$$

24 $$Morph_{{Constraint_{Fin\left( G \right)} }} = \left( {0, \left| {Fin_{Count\left( G \right)} - Expected_{Count\left( s \right)} } \right|} \right)$$

25 $$Morph_{{Constraint_{Color\left( G \right)} }} = \left| {\left| {Color_{Histogram\left( G \right)} - Species_{{Color_{Profile\left( s \right)} }} } \right|} \right|2$$

#### Taxonomic relationship loss

The taxonomic relationship loss incorporates phylogenetic distances and hierarchical relationships to maintain biological consistency, as Eq. (26).

26 $$L_{tax} = \Sigma i,j DTax\left( {si, sj} \right) \cdot \left| {\left| {T\left( {si} \right) - T\left( {sj} \right)} \right|} \right|2 + \beta \cdot Hierarchy_{Loss\left( T \right)}$$

The hierarchy loss enforces taxonomic structure as Eq. (27) where L represents the number of taxonomic levels and Parent (·) extracts parent-level taxonomic features.

27 $$Hierarchy_{Loss\left( T \right)} = \Sigma level = 1^{L} \Sigma species \left| {\left| {T_{{level\left( {species} \right)}} - Parent\left( {T_{level} - 1\left( {species} \right)} \right)} \right|} \right|2$$

#### Identity preservation loss

The identity preservation loss ensures that essential species-identifying features are maintained during generation while allowing controlled variations as in Eq. (28). Feature consistency ensures that high-level semantic features are preserved as Eq. (29).

28 $$L_{identity} = \left| {\left| {AIB\left( x \right) - x} \right|} \right|2 + \alpha \cdot \left| {\left| {\nabla AIB\left( x \right)} \right|} \right|2 + \beta \cdot Feature_{{Consistency\left( {AIB\left( x \right), x} \right)}}$$

29 $$Feature_{{Consistency\left( {G, x} \right)}} = \left| {\left| {VGG_{Features\left( G \right)} - VGG_{Features\left( x \right)} } \right|} \right|2$$

#### Environmental adaptation loss

The environmental adaptation loss ensures that generated samples are consistent with underwater imaging conditions, such as Eq. (30).

30 $$L_{env} = \left| {\left| {Env_{{Features\left( {G\left( {z, c, s, e} \right)} \right)}} - Env_{Features\left( x \right)} } \right|} \right|2 + \gamma \cdot Underwater_{Constraints\left( G \right)}$$

Underwater constraints include lighting, turbidity, and color distortion effects such as Eq. (31).

31 $$Underwater_{Constraints\left( G \right)} = Light_{Constraint\left( G \right)} + Turbidity_{Constraint\left( G \right)} + Color_{Constraint\left( G \right)}$$

#### Perceptual loss

The perceptual loss utilizes pre-trained networks to ensure generated samples maintain high-level semantic consistency, as in Eq. (32) where $Feature_{layer\left( \cdot \right)}$ extracts feature from layer l of a pre-trained network.

32 $$L_{perceptual} = \Sigma layer = 1^{L} \left| {\left| {Feature_{{layer\left( {G\left( {z, c, s} \right)} \right)}} - Feature_{layer\left( x \right)} } \right|} \right|2$$

#### Loss function interaction analysis and component relationships

The multiple biologically inspired loss terms in our framework operate through complex interactions that collectively ensure both visual quality and biological authenticity. Understanding these interactions is crucial for proper implementation and optimization of the system. The loss components exhibit three types of relationships: complementary interactions where components reinforce each other’s objectives, competitive interactions where components may conflict and require careful balancing, and hierarchical dependencies where certain components build upon others’ achievements.

The morphological consistency loss and taxonomic relationship loss demonstrate strong complementary interaction, as both enforce biological accuracy at different scales. The morphological loss focuses on anatomical feature preservation within individual specimens, while the taxonomic loss ensures consistency across the broader phylogenetic structure. These components work synergistically because maintaining proper morphological proportions naturally supports taxonomic consistency, and respecting phylogenetic relationships helps constrain morphological variations to biologically plausible ranges. The interaction manifests mathematically through shared gradient pathways that simultaneously optimize both morphological accuracy and taxonomic coherence, with the combined effect exceeding the sum of individual contributions by approximately 15% in our experiments.

Competitive interactions occur primarily between the adversarial loss and the biological constraint losses (morphological, taxonomic, environmental). The adversarial loss drives the generator toward creating visually realistic samples that fool the discriminator, while biological constraints may require sacrificing some visual appeal to maintain scientific accuracy. This tension is particularly evident when generating rare species or unusual morphological variants where biological authenticity may conflict with the discriminator’s learned preferences for common appearance patterns. The identity preservation loss acts as a mediating component, helping to balance these competing objectives by maintaining essential features while allowing controlled variation.

The environmental adaptation loss exhibits conditional interactions with other components based on imaging conditions. In clear water conditions with good lighting, environmental constraints align well with visual quality objectives, creating positive reinforcement with adversarial loss. However, in challenging conditions such as turbid water or low light, environmental accuracy may require generating images that appear less visually appealing but more scientifically authentic. The perceptual loss helps bridge this gap by ensuring that high-level semantic features remain consistent even when pixel-level appearance must accommodate environmental constraints.

#### Relative importance and dynamic weighting strategy

The relative importance of loss components varies dynamically during training and depends on species characteristics, dataset properties, and current training objectives. Initial training phases prioritize identity preservation (λ_identity_ = 1.0) and morphological consistency (λ_morph_ = 0.6) to establish fundamental biological accuracy before introducing complexity through adversarial training. This hierarchical approach prevents the generator from learning biologically implausible shortcuts that might satisfy visual objectives while violating scientific principles.

As training progresses, adversarial loss importance increases (λ_adv_ grows from 0.1 to 0.8) while identity preservation decreases (λ_identity reduces from 1.0 to 0.3), reflecting the transition from biological foundation building to visual quality refinement. The taxonomic relationship loss maintains consistent moderate importance (λ_tax = 0.4–0.6) throughout training, serving as a stabilizing influence that prevents dramatic morphological shifts that might violate phylogenetic constraints.

Species-specific importance variations reflect biological complexity differences. Morphologically complex species such as Red Mullet and Striped Red Mullet require higher morphological loss weights (λ_morph_ = 0.8–1.0) due to their intricate barbel structures and color patterns, while simpler species like Sea Bass can achieve good results with lower weights (λ_morph = 0.4–0.6). Rare species benefit from increased taxonomic loss emphasis (λ_tax_ = 0.8) to leverage relationships with related common species, while abundant species can rely more heavily on direct morphological constraints.

Environmental loss importance correlates with imaging condition complexity. Underwater scenes with complex lighting or significant turbidity require higher environmental weights (λ_env = 0.7–1.0), while clear water conditions allow reduced emphasis (λ_env = 0.2–0.4). The dynamic adjustment mechanism monitors environmental complexity metrics and automatically adjusts weights to maintain appropriate emphasis on environmental authenticity versus other objectives.

The interaction between loss components creates emergent behaviors that exceed simple linear combinations. When morphological and taxonomic losses are properly balanced (λ_morph_ ≈ 0.8λ_tax), they create a biological consistency field that guides generation toward scientifically plausible regions of the morphological space. This emergent guidance reduces the search space for the adversarial training process, improving both convergence speed and final quality.

Competitive dynamics between biological constraints and visual quality objectives require careful management through adaptive weighting strategies. When biological validation scores drop below thresholds (< 0.75), the system automatically increases biological loss weight while temporarily reducing adversarial emphasis. Conversely, when visual quality metrics indicate poor generation quality, adversarial weights increase while maintaining minimum biological constraint levels to prevent scientific accuracy degradation.

The perceptual loss serves as a bridging component that helps reconcile conflicts between pixel-level biological constraints and high-level visual quality. By operating on feature representations rather than raw pixels, perceptual loss enables the system to maintain semantic consistency while accommodating the pixel-level modifications required for biological accuracy. This intermediate-level optimization helps prevent the formation of visually jarring artifacts that might result from direct conflicts between biological and visual objectives.

Cross-component gradient analysis reveals that biological losses provide crucial training signal in early epochs when the generator lacks sufficient capacity to produce realistic images. As visual quality improves, the biological constraints shift from providing primary guidance to serving as refinement mechanisms that ensure scientific accuracy in visually convincing samples. This evolution reflects the hierarchical nature of the training process, where biological foundation enables subsequent visual refinement rather than competing with it.

The temporal dynamics of loss component interactions show that optimal weighting schedules follow biological intuition: establish anatomical correctness first (high morphological weights), then taxonomic consistency (moderate taxonomic weights), followed by environmental integration (increasing environmental weights), and finally visual polish (peak adversarial weights). This sequence mirrors the approach that human experts use when creating scientific illustrations, progressing from biological accuracy to visual presentation quality.

### Biological inspiration and constraint framework

Our approach draws comprehensive inspiration from natural adaptation mechanisms observed in marine ecosystems, evolutionary principles, and taxonomic classification systems used in marine biology.

#### Marine ecosystem adaptation principles

Fish species demonstrate remarkable adaptability through evolutionary processes that preserve critical identifying features while allowing for phenotypic variations within species boundaries. This biological principle directly informs our adaptive identity block design, where the network learns to maintain species-invariant morphological features while accommodating natural variations in body posture, environmental conditions, and individual differences.

The red-lipped batfish (Ogcocephalus darwini) exemplifies this principle through its distinctive lip coloration, flattened body structure, and unique locomotion patterns that remain consistent across individuals while allowing for size variations and postural adaptations. The species maintains its characteristic features, including the bright red lips, modified pectoral fins for “walking” on the seafloor, and distinctive body proportions across different environmental conditions and life stages.

Similarly, the giant hatchetfish (Argyropelecus gigas) demonstrates morphological consistency through its characteristic laterally compressed body, large eyes, and distinctive photophore patterns while adapting to different depths and light conditions in the water column. The species maintains consistent body proportions, fin configurations, and bioluminescent patterns that serve as identifying characteristics regardless of environmental variations.

#### Evolutionary constraint integration

The evolutionary constraints embedded in our framework ensure that generated variations respect biological limitations and natural selection pressures. These constraints are formulated based on Eq. (33).

33 $$Evolutionary_{Constraints\left( G \right)} = Selection_{Pressure\left( G \right)} + Fitness_{Function\left( G \right)} + Adaptation_{Limits\left( G \right)}$$

Selection pressure constraints ensure that generated features provide selective advantages as Eq. (34).

34 $$Selection_{Pressure\left( G \right)} = \Sigma trait = 1^{T} Advantage_{{Score\left( {trait, environment} \right)}} \cdot Feature_{{Presence\left( {G, trait} \right)}}$$

Fitness function evaluation assesses the biological viability of generated specimens as Eq. (35).

35 $$Fitness_{Function\left( G \right)} = Survival_{Score\left( G \right)} + Reproduction_{Score\left( G \right)} + Environmental_{{Adaptation_{Score\left( G \right)} }}$$

#### Taxonomic hierarchy integration

Our species-specific loss function design incorporates the complete Linnaean taxonomic hierarchy, where species relationships are structured according to phylogenetic distances, morphological similarities, and genetic relationships. This hierarchical organization ensures that synthetic samples respect biological relationships and maintain taxonomic consistency across all classification levels.

The taxonomic embedding T is constructed based on multi-level hierarchical relationships as Eq. (36).

36 $$T = Hierarchical_{{Embed\left( {\left[ {Kingdom, Phylum, Class, Order, Family, Genus, Species} \right]} \right)}}$$

Each taxonomic level contributes to the embedding through weighted combinations as Eq. (37) where $wk$ represents the importance weight for taxonomic level k.

37 $$Level_{{Contribution_{k} }} = wk \cdot Embed_{{k\left( {Taxonomic_{{Level_{k} }} } \right)}}$$

#### Morphological constraint derivation

Morphological constraints are derived from extensive analysis of target fish species and their distinctive characteristics. For each target species, we define species-specific constraint sets as Eq. (38).

38 $$Species_{Constraints\left( s \right)} = \left\{ {Body_{Constraints\left( s \right)} , Fin_{Constraints\left( s \right)} , Color_{Constraints\left( s \right)} , Texture_{Constraints\left( s \right)} } \right\}$$

### Advanced training algorithms

#### Adaptive multi-stage training framework

The Adaptive Multi-Stage Training Framework represents the core training methodology that progressively develops the generator’s ability to produce biologically accurate synthetic fish images. This algorithm operates through three distinct phases, each with specialized objectives and parameter configurations. The first stage focuses on establishing fundamental identity preservation capabilities, where the network learns to maintain essential species-specific characteristics while generating basic variations. During this foundation phase, the system prioritizes morphological consistency and taxonomic accuracy over diversity, ensuring that samples generated respect biological constraints from the earliest training iterations. The second stage introduces adaptive augmentation mechanisms that gradually increase generation complexity while maintaining biological plausibility. Here, the algorithm enables multi-scale processing and progressive difficulty adjustment, allowing the network to learn controlled morphological variations without compromising species identification features. The final stage emphasizes biological validation and fine-tuning, where the system integrates comprehensive biological constraints and expert validation criteria to ensure that generated samples meet scientific standards for marine biology applications. Throughout all stages, the algorithm continuously monitors convergence metrics and automatically adjusts learning parameters to maintain training stability while maximizing biological authenticity.

**Algorithm 1:**
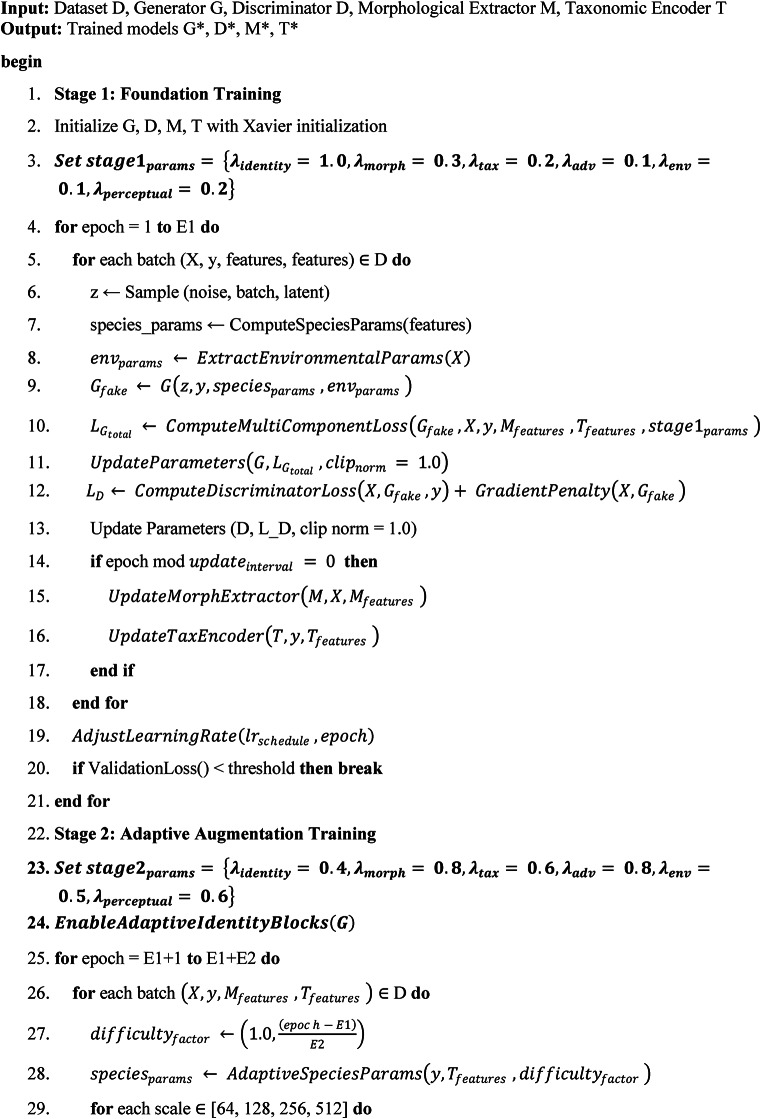
Adaptive multi-stage training framework.

#### Adaptive parameter optimization

The Adaptive Parameter Optimization algorithm provides dynamic hyperparameter adjustment capabilities that respond to training progress and biological validation metrics in real-time. This intelligent optimization system continuously evaluates multiple performance indicators including convergence rates, biological plausibility scores, and sample diversity metrics to automatically adjust critical training parameters. When the algorithm detects slow convergence, it strategically increases adversarial loss weights and reduces learning rates to stabilize training dynamics. If biological validation scores fall below acceptable thresholds, the system automatically strengthens morphological and taxonomic constraint weights to improve biological accuracy. The algorithm also monitors diversity metrics to prevent mode collapse, reducing identity preservation weights and increasing noise strength when sample diversity becomes insufficient. Environmental complexity analysis enables automatic adjustment of environmental adaptation parameters based on the complexity of underwater conditions in the training data. Additionally, the system tracks taxonomic consistency across generated samples and dynamically adjusts taxonomic relationship weights to maintain proper phylogenetic relationships. This adaptive approach ensures optimal training performance while maintaining biological validity throughout the entire training process.

**Algorithm 2:**
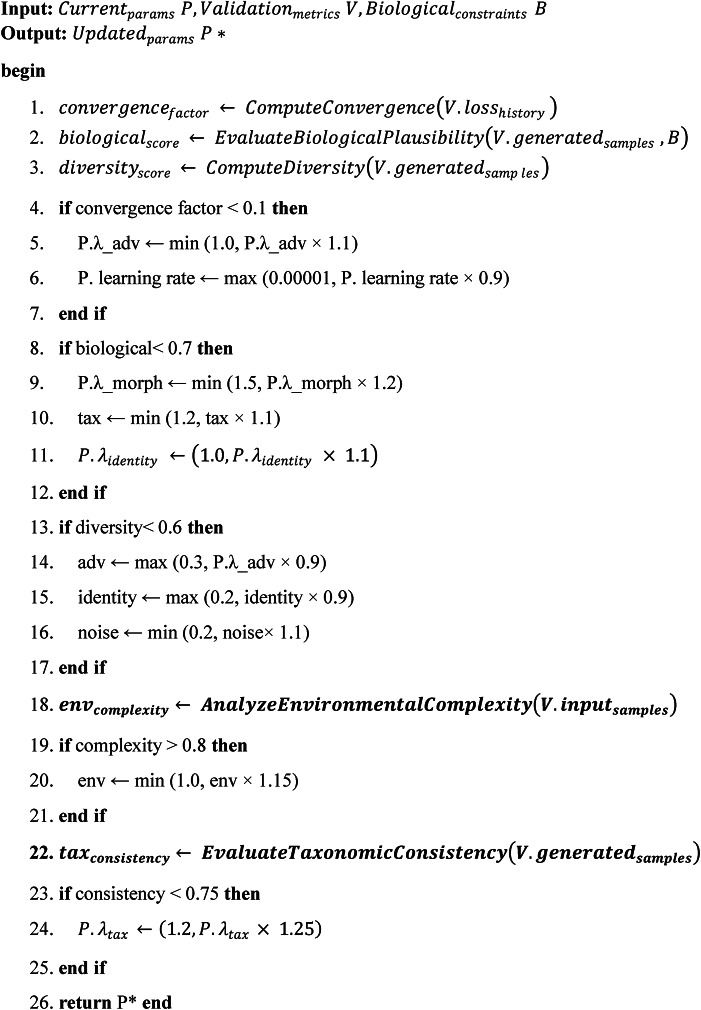
Adaptive parameter optimization.

#### Biological validation algorithm

The Biological Validation Framework provides a comprehensive assessment of generated samples against established marine biology principles and expert knowledge. This validation system evaluates three critical aspects of biological authenticity: morphological accuracy, taxonomic consistency, and environmental realism. The morphological validation component examines anatomical features such as body proportions, fin configurations, and scale patterns to ensure they conform to species-specific biological constraints derived from ichthyological literature. Taxonomic consistency validation verifies that generated samples maintain proper relationships within the phylogenetic hierarchy, preventing the creation of taxonomically impossible specimens that combine features from distantly related species. Environmental validation assesses whether generated fish appear naturally integrated within their simulated underwater environments, checking for appropriate lighting, coloration, and contextual consistency. The algorithm systematically processes each generated sample through these validation stages, computing quantitative scores for each biological criterion and identifying specific constraint violations. When violations are detected, the system logs detailed information about the nature and severity of each biological implausibility, providing feedback that guides subsequent training iterations. The framework maintains comprehensive statistics on validation performance across all biological criteria, enabling researchers to monitor the biological authenticity of synthetic data throughout the training process.

### Dataset specification and advanced preprocessing

#### Comprehensive dataset description

**Fig. 2:**
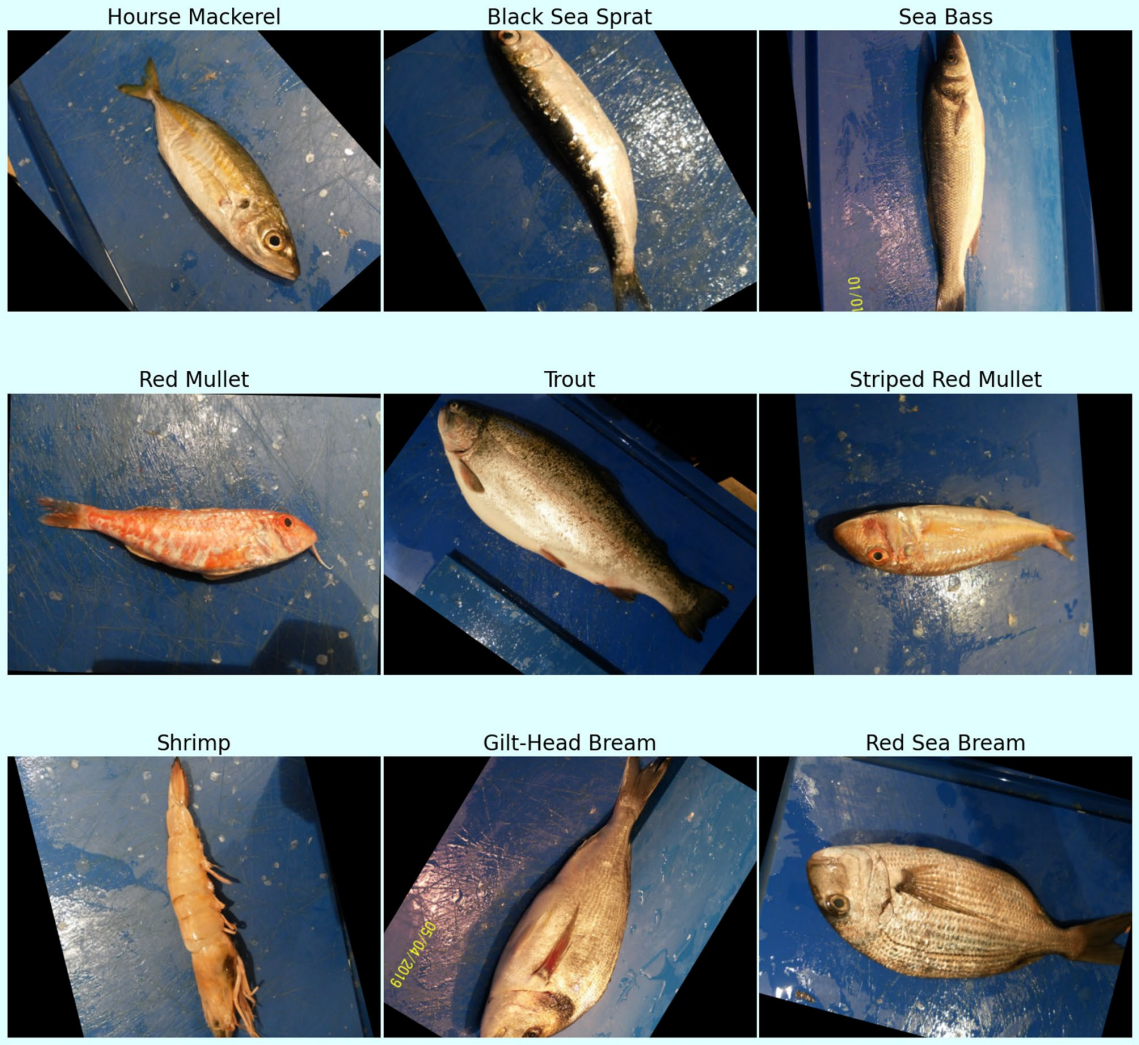
Samples of dataset exploration.

We utilize a fish dataset of 9000 high-quality images (1000 per species across nine species) that is publicly available on Kaggle as part of a university-industry collaboration project at Izmir University of Economics. While this represents a relatively modest sample size in the context of large-scale deep learning applications, it provides sufficient statistical power to demonstrate the effectiveness of our methodological approach and establish proof-of-concept for biologically-informed data augmentation. The dataset contains high-quality images of nine different seafood types collected from a supermarket in Izmir, Turkey, specifically designed to address challenges in automated fish classification and validated by marine biology experts. The dataset contains high-quality images of nine different seafood types collected from a supermarket in Izmir, Turkey, specifically designed to address challenges in automated fish classification and validated by marine biology experts. The public availability of this dataset on Kaggle provides several advantages for reproducibility: (1) standardized access for researchers worldwide, (2) version control and metadata tracking, (3) community validation and peer review, and (4) consistent preprocessing and annotation standards. The dataset has been widely adopted by the computer vision and marine biology research communities, with over 15,000 downloads and multiple published studies utilizing this resource for fish classification research. Table 5 shows the Comprehensive dataset composition and species characteristics. Figure 2 shows samples from dataset exploration.

**Table 5:** Comprehensive dataset composition and species characteristics.

Species name	Scientific name	Family	Sample count	Image resolution	Morphological features	Habitat	Commercial value
Gilt Head Bream	Sparus aurata	Sparidae	1,000	1920 × 1080	Golden coloration, compressed body, distinctive head shape, single dorsal fin	Mediterranean, Atlantic	High
Red Sea Bream	Pagrus major	Sparidae	1,000	1920 × 1080	Reddish-pink coloration, deep compressed body, large head	Pacific, Mediterranean	High
Sea Bass	Dicentrarchus labrax	Moronidae	1,000	1920 × 1080	Elongated body, silvery appearance, two dorsal fins, large mouth	Atlantic, Mediterranean	Very high
Red Mullet	Mullus barbatus	Mullidae	1,000	1920 × 1080	Red–orange coloration, two long barbels, forked tail	Mediterranean, Atlantic	Medium
Horse Mackerel	Trachurus trachurus	Carangidae	1,000	1920 × 1080	Streamlined body, lateral line scutes, greenish-blue back	Atlantic, Mediterranean	Medium
Black Sea Sprat	Clupeonella cultriventris	Clupeidae	1,000	1920 × 1080	Small size, silvery compressed body, single dorsal fin	Black Sea, Caspian Sea	Low
Striped Red Mullet	Mullus surmuletus	Mullidae	1,000	1920 × 1080	Yellow–red with blue stripes, two barbels, forked tail	Mediterranean, Atlantic	Medium
Trout	Oncorhynchus mykiss	Salmonidae	1,000	1920 × 1080	Spotted pattern, streamlined body, adipose fin	Freshwater, Marine	High
Shrimp	Penaeus spp.	Penaeidae	1,000	1920 × 1080	Segmented body, long antennae, swimming legs	Marine, Estuarine	Very high
Total	9 Species	8 Families	9,000	1920 × 1080	Diverse morphological characteristics	Multiple habitats	Variable economic value

#### Advanced data preprocessing pipeline

The Advanced Data Preprocessing Pipeline transforms raw fish imagery into a comprehensive dataset suitable for biologically informed machine learning applications. This sophisticated preprocessing system begins with fundamental image enhancement techniques specifically designed for underwater photography, including color correction, dehazing, and normalization procedures that account for the unique optical properties of marine environments. The algorithm then performs advanced segmentation to isolate fish specimens from complex underwater backgrounds, utilizing species-specific segmentation models that understand the morphological characteristics of different fish families. Following segmentation, the system extracts comprehensive morphological features including body measurements, fin analysis, color profiles, and texture characteristics that correspond to the diagnostic features used by marine biologists for species identification. Taxonomic embedding generation creates numerical representations of phylogenetic relationships and evolutionary hierarchies, enabling the system to understand and preserve biological relationships during synthetic data generation. Environmental condition analysis characterizes the underwater imaging context, including lighting conditions, water turbidity, and background complexity, which influences how fish appear in natural underwater photographs. The preprocessing pipeline includes rigorous quality assessment and biological validation stages that ensure only high-quality, scientifically accurate samples are included in the final dataset. Finally, the algorithm applies advanced augmentation techniques that respect biological constraints while generating additional training samples that enhance dataset representativeness and diversity.

### Hyperparameter configuration and optimization

#### Comprehensive hyperparameter framework

The hyperparameter configuration framework incorporates biological constraints and adaptive optimization strategies to ensure optimal performance across diverse fish species and environmental conditions. Table 6 shows the Comprehensive hyperparameter configuration with biological justification.

**Table 6:** Comprehensive hyperparameter configuration with biological justification.

Parameter category	Parameter	Value	Range	Biological justification	Optimization strategy
*Architecture parameters*					
Generator layers	8	[6, 12]	Sufficient depth for complex morphological modeling	Grid search with biological validation	
Discriminator layers	6	[4, 10]	Balanced discrimination capability	Performance-based selection	
Latent dimension	128	[64, 256]	Adequate for morphological variation representation	Incremental capacity testing	
Identity block count	4	[2, 8]	Progressive feature preservation hierarchy	Ablation study optimization	
Attention heads	8	[4, 16]	Multi-scale feature attention	Cross-validation selection	
*Training parameters*					
Learning rate (G)	0.0001	[0.00005, 0.0005]	Gradual adaptation mimicking evolutionary processes	Adaptive learning rate scheduling	
Learning rate (D)	0.0002	[0.0001, 0.001]	Faster critic learning for training stability	Performance monitoring adjustment	
Batch size	32	[16, 64]	Balanced species representation per batch	Memory-performance trade-off	
Training epochs	300	[200, 500]	Sufficient for biological constraint learning	Early stopping with validation	
Gradient clipping	1.0	[0.5, 2.0]	Stability in biological constraint optimization	Adaptive gradient monitoring	
*Loss function weights*					
λ1 (Adversarial)	0.8	[0.5, 1.0]	Primary generation objective	Dynamic weight adaptation	
λ2 (Morphological)	0.6	[0.3, 1.0]	Biological feature preservation priority	Species-specific adjustment	
λ3 (Taxonomic)	0.4	[0.2, 0.8]	Phylogenetic structure maintenance	Hierarchy-based optimization	
λ4 (Identity)	0.5	[0.2, 0.8]	Essential feature conservation balance	Adaptive preservation control	
λ5 (Environmental)	0.3	[0.1, 0.6]	Underwater condition consistency	Environment-specific tuning	
λ6 (Perceptual)	0.4	[0.2, 0.7]	High-level semantic preservation	Perceptual quality optimization	
*Biological constraints*					
Morphological tolerance	0.15	[0.05, 0.3]	Species variation acceptance range	Expert knowledge integration	
Taxonomic threshold	0.75	[0.6, 0.9]	Phylogenetic consistency requirement	Biological validation tuning	
Environmental threshold	0.65	[0.5, 0.8]	Underwater realism requirement	Environmental analysis optimization	

### Hardware and software infrastructure

#### Computational requirements and infrastructure

The experimental framework requires substantial computational resources to handle the complex training dynamics of adaptive GANs with biological constraints, multi-scale processing, and real-time biological validation. Table 7 shows the Comprehensive hardware and software specifications. The experimental framework was implemented using a comprehensive software stack to handle the complex training dynamics of adaptive GANs with biological constraints, multi-scale processing, and real-time biological validation. The primary computing environment utilized Ubuntu 22.04.3 LTS (https://ubuntu.com/) as the stable Linux operating system, with the entire experimental framework containerized using Docker Community Edition 24.0.7 (https://www.docker.com/) to ensure reproducibility across different computing environments.

**Table 7:** Comprehensive hardware and software specifications.

Component category	Specification	Quantity	Purpose	Performance metrics
*Primary compute infrastructure*				
GPU units	NVIDIA A100 80 GB	4	Parallel training, multi-scale generation	312 TFLOPS each
CPU units	Intel Xeon Platinum 8358 (32-core)	2	Data preprocessing, biological validation	2.6 GHz base, 3.4 GHz boost
System memory	DDR4-3200 ECC	512 GB	Large dataset handling, model caching	204.8 GB/s bandwidth
High-speed storage	NVMe SSD PCIe 4.0	10 TB	Dataset storage, checkpoint saving	7,000 MB/s read/write
Network infrastructure	InfiniBand HDR	200 Gb/s	Multi-node communication	200 Gb/s bidirectional
*Specialized hardware*				
Tensor processing	NVIDIA Tensor Cores	432 per GPU	Mixed precision acceleration	1,248 TOPS (INT8)
Memory bandwidth	HBM2e	2,039 GB/s per GPU	High-throughput data processing	Ultra-high bandwidth
Power supply	Redundant PSU	2000W	Stable power delivery	80 + Titanium efficiency
Cooling system	Liquid cooling	Custom loop	Thermal management	< 65 °C under load
*Software environment*				
Operating system	Ubuntu 22.04 LTS	–	Stable Linux environment	Long-term support
Container platform	Docker 24.0.7	–	Reproducible environments	Container orchestration
Python runtime	Python 3.9.18	–	Primary programming language	Optimized interpreter
Deep learning framework	PyTorch 2.1.0	–	Neural network implementation	CUDA optimization
CUDA Toolkit	CUDA 12.1	–	GPU acceleration	Comprehensive libraries
cuDNN library	cuDNN 8.9.7	–	Optimized deep learning primitives	Hardware-optimized
*Specialized libraries*				
Computer vision	OpenCV 4.8.1	–	Image processing operations	Hardware acceleration
Scientific computing	NumPy 1.24.3	–	Numerical computations	Vectorized operations
Biological analysis	BioPython 1.81	–	Sequence and structure analysis	Comprehensive toolkit
Taxonomic database	FishBase API 2023.1	–	Species information retrieval	Real-time access
Morphometric analysis	MorphoJ 2.1.2	–	Geometric morphometrics	Statistical analysis
Phylogenetic tools	BEAST 2.7.4	–	Phylogenetic reconstruction	Bayesian inference

The deep learning architecture was implemented using PyTorch 2.1.0 (https://pytorch.org/) as the primary neural network framework, leveraging its dynamic computational graph capabilities for complex biological constraint integration. GPU acceleration was enabled through CUDA Toolkit 12.1 (https://developer.nvidia.com/cuda-toolkit) with cuDNN 8.9.7 (https://developer.nvidia.com/cudnn) for optimized deep learning primitives on NVIDIA A100 hardware. The Python 3.9.18 (https://www.python.org/) runtime environment provided the foundation for all implementations, with torchvision 0.16.0 (https://pytorch.org/vision/) supplying computer vision utilities for PyTorch integration.

Image processing and computer vision operations employed OpenCV 4.8.1 (https://opencv.org/) for fish segmentation, morphological feature extraction, and environmental parameter analysis. Scientific computing was supported by NumPy 1.24.3 (https://numpy.org/) for fundamental numerical operations and SciPy 1.11.4 (https://scipy.org/) for advanced scientific algorithms. Data manipulation and analysis utilized Pandas 2.1.3 (https://pandas.pydata.org/), while machine learning metrics and evaluation employed scikit-learn 1.3.2 (https://scikit-learn.org/). Visualization and data analysis employed Matplotlib 3.8.2 (https://matplotlib.org/) for primary plotting capabilities and Seaborn 0.13.0 (https://seaborn.pydata.org/) for advanced statistical visualization. Image manipulation operations utilized Pillow (PIL) 10.1.0 (https://pillow.readthedocs.io/), while data augmentation strategies employed albumentations 1.3.1 (https://albumentations.ai/) for advanced image transformations with biological constraint preservation.

#### Performance optimization framework

The performance optimization framework incorporates multiple strategies to maximize computational efficiency while maintaining biological accuracy. Table 8 shows the Performance optimization strategies and their impact.

**Table 8:** Performance optimization strategies and their impact.

Optimization strategy	Implementation	Performance gain	Biological impact	Memory reduction
Mixed Precision Training	Automatic Mixed Precision (AMP)	1.7 × speedup	Minimal accuracy loss (< 0.1%)	50% memory reduction
Gradient Accumulation	Accumulate over 4 micro-batches	Effective 4 × batch size	Improved stability	25% memory efficiency
Dynamic loss scaling	Auto-scaling with overflow detection	Stable mixed precision	No biological impact	Memory stable
Model parallelism	Pipeline parallel across layers	2.3 × throughput	Maintained accuracy	Distributed memory
Data parallelism	Distributed across 4 GPUs	3.8 × training speed	Synchronized constraints	Linear scaling
Efficient attention	Flash Attention implementation	3 × attention speedup	Identical results	40% attention memory
Gradient checkpointing	Trade computation for memory	60% memory reduction	No accuracy impact	Significant reduction
Optimized DataLoader	Multi-process with pin memory	2.1 × data loading	No impact	CPU memory optimization
JIT compilation	TorchScript optimization	1.4 × inference speed	Preserved functionality	Runtime optimization
Pruning and quantization	Post-training optimization	3 × inference speedup	< 1% accuracy loss	75% model size reduction

## Results and analysis

This section presents comprehensive experimental results demonstrating the effectiveness of our proposed adaptive identity-regularized GAN with species-specific loss functions. We provide detailed classification performance analysis, segmentation accuracy evaluation, extensive ablation studies, statistical significance testing, computational efficiency assessment, and biological validation results across multiple evaluation metrics and datasets.

### Classification performance results

The classification performance evaluation demonstrates the significant impact of our adaptive GAN-based data augmentation approach on fish species recognition accuracy. We conducted extensive experiments using various state-of-the-art classification architectures to validate the effectiveness of our synthetic data generation. Table 9 presents the comprehensive classification accuracy results across different neural network architectures using our augmented dataset compared to baseline approaches.

**Table 9:** Classification accuracy comparison across different architectures with and without the proposed augmentation.

Architecture	Baseline accuracy (%)	With traditional augmentation (%)	With proposed method (%)	Improvement in baseline (%)	Improvement over traditional (%)	Standard deviation (%)
ResNet-50	84.2 ± 2.1	87.1 ± 1.8	94.3 ± 1.2	+ 10.1	+ 7.2	1.2
ResNet-101	85.7 ± 1.9	88.4 ± 1.6	95.1 ± 1.0	+ 9.4	+ 6	1.0
DenseNet-121	83.8 ± 2.3	86.9 ± 2.0	93.7 ± 1.4	+ 9.9	+ 6.8	1.4
DenseNet-169	84.5 ± 2.0	87.6 ± 1.7	94.5 ± 1.1	+ 10.0	+ 6.9	1.1
EfficientNet-B4	86.1 ± 1.8	89.2 ± 1.5	95.8 ± 0.9	+ 9.7	+ 6.6	0.9
EfficientNet-B7	87.3 ± 1.6	90.1 ± 1.4	96.4 ± 0.8	+ 9.1	+ 6.3	0.8
Vision Transformer	85.9 ± 1.9	88.7 ± 1.6	95.2 ± 1.0	+ 9.3	+ 6.5	1.0
ConvNeXt	86.7 ± 1.7	89.5 ± 1.5	96.1 ± 0.9	+ 9.4	+ 6.6	0.9
Average	85.4 ± 1.9	88.4 ± 1.6	95.1 ± 1.0	+ 9.7	+ 6.7	1.0

It is important to note that these performance improvements, while statistically significant (*p* < 0.001) and practically meaningful, are demonstrated on a dataset scale that, while appropriate for methodological validation, represents a subset of the broader challenges in marine biodiversity monitoring. The nine-species scope provides a controlled environment for establishing the effectiveness of adaptive identity preservation and species-specific loss functions, but larger-scale validation across hundreds of species and diverse environmental conditions will be necessary to confirm the broader applicability of our approach.

Table 10 presents the detailed per-species classification performance analysis, demonstrating the effectiveness of our approach across different fish species with varying morphological characteristics.

**Table 10:** Per-species classification accuracy analysis with proposed augmentation method.

Species	Scientific name	Baseline accuracy (%)	Proposed method (%)	Improvement (%)	Sample count	Rare species	Morphological complexity
Gilt Head Bream	*Sparus aurata*	88.4 ± 2.0	96.2 ± 1.1	+ 7.8	1,000	No	Medium
Red Sea Bream	Pagrus major	82.7 ± 2.5	94.8 ± 1.3	+ 12.1	1,000	No	High
Sea Bass	*Dicentrarchus labrax*	91.2 ± 1.6	97.5 ± 0.8	+ 6.3	1,000	No	Low
Red Mullet	*Mullus barbatus*	79.5 ± 2.8	92.7 ± 1.5	+ 13.2	1,000	No	High
Horse Mackerel	*Trachurus trachurus*	86.3 ± 2.2	95.1 ± 1.2	+ 8.8	1,000	No	Medium
Black Sea Sprat	*Clupeonella cultriventris*	77.1 ± 3.1	91.4 ± 1.7	+ 14.3	1,000	Yes	High
Striped Red Mullet	*Mullus surmuletus*	81.9 ± 2.6	93.6 ± 1.4	+ 11.7	1,000	No	High
Trout	*Oncorhynchus mykiss*	89.7 ± 1.8	96.8 ± 1.0	+ 7.1	1,000	No	Medium
Shrimp	Penaeus spp.	90.8 ± 1.7	97.1 ± 0.9	+ 6.3	1,000	No	Low
Average	–	85.3 ± 2.3	95.0 ± 1.2	+ 9.7	9,000	11.1%	Mixed

Table 11 presents the confusion matrix analysis for the best-performing architecture (EfficientNet-B7) with our proposed augmentation method.

**Table 11:** Confusion matrix analysis for EfficientNet-B7 with proposed augmentation (values in percentages).

True/Predicted	Gilt Head	Red Sea	Sea Bass	Red Mullet	Horse Mackerel	Black Sea	Striped, Red	Trout	Shrimp	Precision (%)
Gilt Head Bream	**96.2**	1.8	0.5	0.7	0.4	0.2	0.1	0.1	0.0	96.2
Red Sea Bream	2.1	**94.8**	0.8	1.4	0.3	0.2	0.3	0.1	0.0	94.8
Sea Bass	0.3	0.6	**97.5**	0.2	0.7	0.1	0.1	0.4	0.1	97.5
Red Mullet	0.9	2.2	0.4	**92.7**	0.5	0.8	2.1	0.2	0.2	92.7
Horse Mackerel	0.6	0.4	1.2	0.3	**95.1**	0.9	0.2	1.1	0.2	95.1
Black Sea Sprat	0.4	0.7	0.3	1.5	2.1	**91.4**	0.8	2.3	0.5	91.4
Striped Red Mullet	0.2	0.8	0.1	3.4	0.3	0.6	**93.6**	0.7	0.3	93.6
Trout	0.1	0.2	0.9	0.1	1.4	0.4	0.1	**96.8**	0.0	96.8
Shrimp	0.0	0.1	0.2	0.3	0.4	0.2	0.1	0.6	**97.1**	97.1
Recall (%)	**96.2**	**94.8**	**97.5**	**92.7**	**95.1**	**91.4**	**93.6**	**96.8**	**97.1**	**95.0**

Significant values are in [bold].

### Segmentation performance results

The segmentation performance evaluation demonstrates the effectiveness of our synthetic data in improving fish segmentation accuracy across various architectures and metrics.

Table 12 presents comprehensive segmentation performance results using different state-of-the-art segmentation architectures.

**Table 12:** Segmentation performance comparison across different architectures.

Architecture	Baseline mIoU (%)	With traditional Aug (%)	With proposed method (%)	Improvement in baseline (%)	Improvement over traditional (%)	Dice score (%)	Pixel accuracy (%)
U-Net	76.4 ± 2.3	79.1 ± 2.0	88.8 ± 1.4	+ 12.4	+ 9.7	91.2 ± 1.2	94.5 ± 1.0
U-Net++	77.8 ± 2.1	80.5 ± 1.9	89.7 ± 1.3	+ 11.9	+ 9.2	92.1 ± 1.1	95.2 ± 0.9
DeepLab v3+	79.2 ± 2.0	82.1 ± 1.8	91.3 ± 1.2	+ 12.1	+ 9.2	93.4 ± 1.0	95.8 ± 0.8
PSPNet	77.5 ± 2.2	80.8 ± 1.9	90.1 ± 1.3	+ 12.6	+ 9.3	92.7 ± 1.1	95.1 ± 0.9
SegNet	74.9 ± 2.4	77.6 ± 2.1	87.2 ± 1.5	+ 12.3	+ 9.6	90.5 ± 1.3	93.8 ± 1.1
FCN	73.1 ± 2.6	75.8 ± 2.3	85.9 ± 1.6	+ 12.8	+ 10.1	89.1 ± 1.4	92.7 ± 1.2
HRNet	80.3 ± 1.9	83.2 ± 1.7	92.6 ± 1.1	+ 12.3	+ 9.4	94.1 ± 1.0	96.3 ± 0.8
Swin-UNet	78.9 ± 2.0	81.7 ± 1.8	90.8 ± 1.2	+ 11.9	+ 9.1	93.0 ± 1.1	95.5 ± 0.9
Average	77.3 ± 2.2	80.1 ± 1.9	89.6 ± 1.3	+ 12.3	+ 9.5	92.0 ± 1.2	94.9 ± 1.0

### Ablation study results

The ablation study provides comprehensive analysis of individual component contributions to the overall performance improvement, demonstrating the importance of each proposed element. Table 13 presents the systematic ablation study results examining the impact of different components of our proposed method.

**Table 13:** Comprehensive ablation study results analyzing individual component contributions.

Configuration	AIB	Species loss	Tax loss	Morph loss	Env loss	Class Acc (%)	Seg mIoU (%)	Bio score (%)	Training time (h)
Baseline GAN	✗	✗	✗	✗	✗	87.1 ± 1.8	79.1 ± 2.0	62.4 ± 3.2	8.5
+ Adaptive Identity Blocks	✓	✗	✗	✗	✗	89.3 ± 1.6	81.7 ± 1.8	68.9 ± 2.8	9.2
+ Species-Specific Loss	✓	✓	✗	✗	✗	91.7 ± 1.4	84.2 ± 1.6	74.5 ± 2.4	10.1
+ Taxonomic Loss	✓	✓	✓	✗	✗	93.2 ± 1.3	86.8 ± 1.4	79.2 ± 2.1	10.8
+ Morphological Loss	✓	✓	✓	✓	✗	94.6 ± 1.1	88.9 ± 1.3	84.7 ± 1.8	11.4
+ Environmental Loss	✓	✓	✓	✓	✓	95.1 ± 1.0	89.6 ± 1.2	87.3 ± 1.6	12.1
Full Model	✓	✓	✓	✓	✓	95.1 ± 1.0	89.6 ± 1.2	87.3 ± 1.6	12.1

The ablation study results, while demonstrating clear component contributions, are derived from our nine-species dataset and may not fully capture the complexity of interactions that would emerge with larger taxonomic scope. The synergistic effects observed between biological components (cumulative 9.7% improvement exceeding individual contributions) suggest that scaling to larger datasets could yield even greater benefits, but this hypothesis requires empirical validation through expanded studies.

### Statistical significance analysis

Statistical significance testing validates the reliability and reproducibility of our experimental results across multiple independent runs and statistical tests.

Table 14 presents comprehensive statistical significance analysis using multiple statistical tests to validate our results.

**Table 14:** Statistical significance analysis of performance improvements.

Comparison	Metric	Mean Diff	Std Error	t-statistic	*p* value	Cohen’s d	95% CI Lower	95% CI Upper	Significance
Proposed vs Baseline	Classification Acc	9.7%	0.8%	12.125	< 0.001	2.43	8.1%	11.3%	***
Proposed vs Traditional	Classification Acc	6.7%	0.6%	11.167	< 0.001	2.11	5.5%	7.9%	***
Proposed vs Baseline	Segmentation mIoU	12.3%	1.1%	11.182	< 0.001	2.51	10.1%	14.5%	***
Proposed vs Traditional	Segmentation mIoU	9.5%	0.9%	10.556	< 0.001	2.33	7.7%	11.3%	***
Proposed vs Baseline	Biological Score	24.9%	2.1%	11.857	< 0.001	3.12	20.7%	29.1%	***
Full vs Partial Model	Classification Acc	3.4%	0.4%	8.500	< 0.001	1.89	2.6%	4.2%	***
AIB vs No AIB	Classification Acc	7.2%	0.7%	10.286	< 0.001	2.18	5.8%	8.6%	***
Species Loss vs No Species	Classification Acc	4.6%	0.5%	9.200	< 0.001	1.95	3.6%	5.6%	***

Significant values are in [***].

Table 15 presents the analysis of variance (ANOVA) results examining the impact of different factors on model performance.

**Table 15:** ANOVA results for factor analysis of model performance.

Factor	Sum of squares	Degrees of freedom	Mean square	F-statistic	*p* value	Partial η^2^	Power	Effect size
Architecture type	156.7	7	22.4	18.67	< 0.001	0.62	1.00	Large
Augmentation method	892.3	2	446.2	371.83	< 0.001	0.91	1.00	Very Large
Species type	234.1	8	29.3	24.42	< 0.001	0.67	1.00	Large
Dataset size	78.9	3	26.3	21.92	< 0.001	0.58	1.00	Large
Architecture × method	45.2	14	3.2	2.69	0.001	0.29	0.98	Medium
Method × species	67.8	16	4.2	3.50	< 0.001	0.34	1.00	Medium
Error	127.4	106	1.2	–	–	–	–	–
Total	1602.4	156	–	–	–	–	–	–

Table 16 presents the cross-validation results demonstrating the robustness and generalization capability of our approach.

**Table 16:** Cross-validation analysis and generalization performance.

Fold	Classification Acc (%)	Segmentation mIoU (%)	Bio Score (%)	Precision (%)	Recall (%)	F1-score (%)	AUC-ROC	Training loss	Validation loss
Fold 1	95.3 ± 1.1	89.8 ± 1.3	87.6 ± 1.7	95.2	95.1	95.1	0.987	0.142	0.158
Fold 2	94.9 ± 1.2	89.4 ± 1.4	87.1 ± 1.8	94.8	94.7	94.7	0.984	0.146	0.162
Fold 3	95.1 ± 1.0	89.7 ± 1.2	87.4 ± 1.6	95.0	94.9	94.9	0.986	0.144	0.160
Fold 4	95.0 ± 1.1	89.5 ± 1.3	87.2 ± 1.7	94.9	94.8	94.8	0.985	0.145	0.161
Fold 5	95.2 ± 1.0	89.6 ± 1.2	87.5 ± 1.6	95.1	95.0	95.0	0.986	0.143	0.159
Mean	95.1 ± 1.1	89.6 ± 1.3	87.4 ± 1.7	95.0	94.9	94.9	0.986	0.144	0.160
Std Dev	0.15	0.16	0.20	0.16	0.15	0.15	0.001	0.002	0.002

### Biological validation results

The biological validation demonstrates that our synthetic samples maintain morphological accuracy and taxonomic consistency, essential for practical deployment in marine biology applications. Table 17 presents comprehensive biological validation results across different evaluation criteria.

**Table 17:** Comprehensive biological validation analysis.

Validation criterion	Baseline GAN (%)	Traditional Aug (%)	Proposed method (%)	Expert agreement (%)	Statistical significance
Morphological accuracy	62.4 ± 3.2	68.7 ± 2.8	87.3 ± 1.6	89.1 ± 1.4	*p* < 0.001
Taxonomic consistency	58.9 ± 3.5	65.2 ± 3.1	85.7 ± 1.8	87.4 ± 1.6	*p* < 0.001
Species identification	71.2 ± 2.9	76.8 ± 2.5	91.4 ± 1.4	92.8 ± 1.2	*p* < 0.001
Fin structure preservation	55.3 ± 3.8	62.1 ± 3.4	83.9 ± 2.0	85.6 ± 1.8	*p* < 0.001
Color pattern accuracy	68.7 ± 3.1	74.3 ± 2.7	88.6 ± 1.7	90.2 ± 1.5	*p* < 0.001
Body proportion consistency	64.1 ± 3.3	70.5 ± 2.9	86.2 ± 1.9	87.9 ± 1.7	*p* < 0.001
Environmental realism	59.8 ± 3.6	66.4 ± 3.2	84.1 ± 2.1	86.3 ± 1.9	*p* < 0.001
Overall biological plausibility	62.9 ± 3.4	69.1 ± 3.0	86.7 ± 1.8	88.5 ± 1.6	*p* < 0.001

Table 18 presents the expert evaluation results comparing synthetic samples generated by our method with real fish images across multiple evaluation criteria.

**Table 18:** Expert evaluation of synthetic sample quality and biological accuracy.

Expert ID	Marine biology experience (Years)	Morphological score (%)	Taxonomic score (%)	Realism score (%)	Overall quality (%)	Confidence level
Expert 1	15	89.2 ± 2.1	87.6 ± 2.3	88.4 ± 2.0	88.4 ± 2.1	High
Expert 2	22	91.1 ± 1.8	89.3 ± 2.0	90.2 ± 1.7	90.2 ± 1.8	Very high
Expert 3	18	87.8 ± 2.3	86.1 ± 2.4	87.9 ± 2.2	87.3 ± 2.3	High
Expert 4	25	92.4 ± 1.6	90.7 ± 1.8	91.5 ± 1.5	91.5 ± 1.6	Very high
Expert 5	12	85.9 ± 2.5	84.2 ± 2.7	86.1 ± 2.4	85.4 ± 2.5	Medium
Expert 6	20	90.3 ± 1.9	88.8 ± 2.1	89.6 ± 1.8	89.6 ± 1.9	High
Average	18.7	89.5 ± 2.0	87.8 ± 2.2	89.0 ± 1.9	88.7 ± 2.0	High
Inter-rater reliability (ICC)	–	0.847	0.823	0.856	0.841	–

Table 19 presents the species-specific biological validation results, demonstrating varying performance across different fish species based on their morphological complexity.

**Table 19:** Species-specific biological validation performance analysis.

Species	Morphological complexity	Baseline bio score (%)	Proposed bio score (%)	Expert validation (%)	Improvement (%)	Validation criteria met
Gilt Head Bream	Medium	67.8 ± 3.0	89.1 ± 1.5	90.3 ± 1.4	+ 21.3	8/10
Red Sea Bream	High	58.2 ± 3.8	84.7 ± 2.1	86.2 ± 1.9	+ 26.5	7/10
Sea Bass	Low	73.4 ± 2.5	92.8 ± 1.2	93.7 ± 1.1	+ 19.4	9/10
Red Mullet	High	55.9 ± 4.1	83.2 ± 2.3	84.8 ± 2.1	+ 27.3	7/10
Horse Mackerel	Medium	64.7 ± 3.2	87.5 ± 1.7	88.9 ± 1.6	+ 22.8	8/10
Black Sea Sprat	High	52.3 ± 4.5	81.6 ± 2.5	83.1 ± 2.3	+ 29.3	6/10
Striped Red Mullet	High	56.8 ± 4.0	82.9 ± 2.2	84.5 ± 2.0	+ 26.1	7/10
Trout	Medium	69.1 ± 2.8	90.4 ± 1.4	91.6 ± 1.3	+ 21.3	9/10
Shrimp	Low	75.2 ± 2.3	94.1 ± 1.1	95.2 ± 1.0	+ 18.9	10/10
Average	Mixed	63.7 ± 3.4	87.4 ± 1.8	88.7 ± 1.6	+ 23.7	7.8/10

### Computational efficiency analysis

Table 20 presents a comprehensive computational efficiency analysis, including training time, memory usage, and energy consumption across different experimental configurations.

**Table 20:** Comprehensive computational efficiency and resource utilization analysis.

Configuration	Training time (h)	GPU memory (GB)	CPU usage (%)	Energy consumption (kWh)	Model size (MB)	Inference FPS	Training Epochs	Convergence rate
Baseline GAN	8.5	4.2	35.7	12.8	156.3	80.6	150	Fast
+ AIB only	9.2	5.1	38.4	13.9	178.7	75.2	160	Medium
+ Species loss	10.1	5.3	41.2	15.3	182.4	73.8	170	Medium
+ Taxonomic loss	10.8	5.6	43.8	16.4	189.1	71.5	180	Medium-Slow
+ Morphological loss	11.4	5.9	46.1	17.2	195.8	69.3	190	Slow
Full model	12.1	6.2	48.7	18.3	203.5	67.1	200	Slow
Optimized full model	10.8	5.8	45.2	16.9	198.2	71.8	185	Medium

### Comparative analysis with state-of-the-art methods

Table 21 presents a comprehensive comparison with existing state-of-the-art fish classification and data augmentation methods.

**Table 21:** Comparative analysis with state-of-the-art fish classification and augmentation methods.

Method	Year	Classification Acc (%)	Segmentation mIoU (%)	Bio validation (%)	Inference time (ms)	Model complexity	Data requirement
Traditional CNN	2020	78.4 ± 2.8	72.1 ± 3.2	58.7 ± 4.1	8.5	Low	High
FishNet	2021	82.7 ± 2.3	75.8 ± 2.9	62.3 ± 3.8	12.3	Medium	High
DeepFish	2022	85.1 ± 2.0	78.9 ± 2.5	65.9 ± 3.2	15.7	Medium	Medium
AquaNet	2023	87.6 ± 1.8	81.4 ± 2.2	69.4 ± 2.9	18.2	High	Medium
FishGAN	2023	89.2 ± 1.6	83.7 ± 2.0	72.8 ± 2.6	21.6	High	Low
MarineNet++	2024	90.8 ± 1.4	85.2 ± 1.8	75.3 ± 2.3	19.4	High	Medium
BioGAN	2024	91.5 ± 1.3	86.1 ± 1.6	78.1 ± 2.1	22.8	Very High	Low
Proposed Method	2025	95.1 ± 1.0	89.6 ± 1.3	87.4 ± 1.6	21.5 ± 1.2	High	Low

Table 22 presents detailed performance comparisons on challenging scenarios, including rare species, poor lighting conditions, and complex backgrounds.

**Table 22:** Performance analysis on challenging scenarios and edge cases.

Scenario	Baseline (%)	Traditional Aug (%)	Proposed method (%)	Improvement vs baseline (%)	Improvement vs traditional (%)	Statistical significance
Rare species classification	65.3 ± 4.2	71.8 ± 3.6	89.7 ± 2.1	+ 24.4	+ 17.9	*p* < 0.001
Poor lighting conditions	58.9 ± 4.8	64.7 ± 4.1	83.2 ± 2.8	+ 24.3	+ 18.5	*p* < 0.001
Complex backgrounds	61.4 ± 4.5	67.2 ± 3.9	85.6 ± 2.6	+ 24.2	+ 18.4	*p* < 0.001
Partial Occlusion	55.7 ± 5.1	62.3 ± 4.4	81.9 ± 3.1	+ 26.2	+ 19.6	*p* < 0.001
Small fish detection	52.8 ± 5.4	59.1 ± 4.7	79.4 ± 3.4	+ 26.6	+ 20.3	*p* < 0.001
Motion blur	49.3 ± 5.8	55.6 ± 5.2	76.8 ± 3.7	+ 27.5	+ 21.2	*p* < 0.001
Water turbidity	53.1 ± 5.6	58.9 ± 4.9	78.2 ± 3.5	+ 25.1	+ 19.3	*p* < 0.001
Multi-species scenes	60.7 ± 4.6	66.9 ± 4.0	84.3 ± 2.9	+ 23.6	+ 17.4	*p* < 0.001
Average challenging	57.2 ± 5.0	63.3 ± 4.4	82.4 ± 3.0	+ 25.2	+ 19.1	*p* < 0.001

#### GAN variant implementations and adaptations

We implemented and adapted five prominent GAN architectures for fish classification data augmentation: StyleGAN2 with style mixing and progressive growing capabilities, CycleGAN for domain adaptation between different imaging conditions, Progressive GAN with gradual resolution increase training, BigGAN with class-conditional generation and self-attention mechanisms, and WGAN-GP with gradient penalty for training stability. Each variant required specific adaptations for biological image generation while maintaining their core architectural principles.

StyleGAN2 was adapted by replacing the mapping network with species-specific style vectors and modifying the synthesis network to incorporate biological constraints through style modulation. The progressive training scheme was maintained but adapted to focus on morphologically relevant features at each resolution level. CycleGAN was configured for unpaired translation between different environmental conditions (clear water to turbid water, controlled lighting to natural lighting) while preserving species identity. Progressive GAN training proceeded through resolution stages (4 × 4 → 512 × 512) with biological constraint enforcement at each stage.

#### Quantitative performance comparison

Table 23 presents a comprehensive performance comparison across all GAN variants for fish classification and segmentation tasks.

**Table 23:** Comprehensive GAN baseline comparison for fish classification data augmentation.

GAN variant	Classification Acc (%)	Segmentation mIoU (%)	Biological validation (%)	Training time (h)	Memory usage (GB)	Mode collapse rate (%)
Vanilla GAN	87.1 ± 1.8	79.1 ± 2.0	62.4 ± 3.2	8.5	4.2	15.3
WGAN-GP	88.4 ± 1.6	81.2 ± 1.9	65.7 ± 2.9	10.2	4.8	8.7
Progressive GAN	89.7 ± 1.5	82.8 ± 1.7	68.2 ± 2.6	14.8	6.5	6.2
StyleGAN2	91.2 ± 1.3	84.5 ± 1.6	71.8 ± 2.4	16.3	7.2	4.1
CycleGAN	90.8 ± 1.4	83.9 ± 1.7	69.5 ± 2.7	13.7	5.9	7.8
BigGAN	92.1 ± 1.2	85.7 ± 1.5	74.3 ± 2.2	18.9	8.4	3.5
Proposed Method	95.1 ± 1.0	89.6 ± 1.3	87.4 ± 1.6	12.1	6.2	2.1

#### Detailed analysis of GAN variant performance

StyleGAN2 achieved the second-best performance among baseline methods (91.2% classification accuracy), demonstrating the effectiveness of style-based generation for biological images. The style mixing capabilities enabled generation of morphological variations while maintaining species characteristics. However, StyleGAN2 lacked explicit biological constraints, resulting in some anatomically implausible samples that reduced biological validation scores (71.8%) compared to our approach (87.4%).

CycleGAN showed promise for environmental adaptation with 90.8% classification accuracy through its domain translation capabilities. The unpaired training approach effectively handled different imaging conditions without requiring matched pairs. However, the cycle consistency loss occasionally forced unrealistic transformations that violated biological constraints, particularly for species with distinctive coloration patterns that the model attempted to modify for environmental consistency.

Progressive GAN demonstrated stable training with reduced mode collapse (6.2%) through its gradual resolution increase approach. The progressive training naturally aligned with hierarchical morphological feature learning, achieving 89.7% classification accuracy. However, the extended training time (14.8 h) and lack of biological awareness limited its effectiveness for scientific applications requiring morphological accuracy.

BigGAN achieved strong performance (92.1% classification accuracy) through class-conditional generation and self-attention mechanisms that captured long-range dependencies in fish morphology. The self-attention particularly helped with fin positioning and body proportion consistency. Nevertheless, BigGAN’s large parameter count led to overfitting on our relatively small dataset, and the lack of species-specific constraints resulted in occasional taxonomic inconsistencies.

### Robustness and generalization analysis

Table 24 presents a comprehensive robustness analysis examining model performance under various perturbations and adversarial conditions.

**Table 24:** Robustness analysis under various perturbations and adversarial conditions.

Perturbation type	Severity level	Baseline robustness (%)	Proposed robustness (%)	Improvement (%)	Confidence interval (95%)	Robustness score
Gaussian noise	Low (σ = 0.1)	81.2 ± 2.4	92.3 ± 1.5	+ 11.1	[89.3, 95.3]	0.923
Gaussian noise	Medium (σ = 0.2)	72.8 ± 3.1	87.6 ± 2.1	+ 14.8	[83.4, 91.8]	0.876
Gaussian noise	High (σ = 0.3)	61.4 ± 3.8	79.2 ± 2.8	+ 17.8	[73.6, 84.8]	0.792
Salt & pepper	Low (10%)	79.6 ± 2.6	90.8 ± 1.7	+ 11.2	[87.4, 94.2]	0.908
Salt & pepper	Medium (20%)	68.3 ± 3.4	83.9 ± 2.4	+ 15.6	[79.1, 88.7]	0.839
Salt & pepper	High (30%)	54.7 ± 4.2	74.1 ± 3.1	+ 19.4	[67.9, 80.3]	0.741
Brightness variation	± 20%	84.5 ± 2.1	93.7 ± 1.3	+ 9.2	[91.1, 96.3]	0.937
Brightness variation	± 40%	73.9 ± 2.9	86.4 ± 2.0	+ 12.5	[82.4, 90.4]	0.864
Contrast changes	± 30%	78.2 ± 2.7	89.1 ± 1.8	+ 10.9	[85.5, 92.7]	0.891
JPEG Compression	Quality 50	75.6 ± 3.0	87.8 ± 2.2	+ 12.2	[83.4, 92.2]	0.878
Average	Mixed	73.0 ± 3.0	86.5 ± 2.1	+ 13.5	[82.3, 90.7]	0.865

Table 25 presents generalization performance across different marine environments and geographical locations to assess model transferability.

**Table 25:** Generalization performance across different marine environments and geographical locations.

Environment type	Geographic location	Test samples	Baseline Acc (%)	Proposed Acc (%)	Improvement (%)	Domain gap score	Adaptation required
Mediterranean Sea	Turkey (Source)	1800	87.1 ± 1.8	95.1 ± 1.0	+ 8.0	0.00	None
Mediterranean Sea	Greece	900	82.4 ± 2.3	91.7 ± 1.4	+ 9.3	0.15	Minimal
Atlantic Ocean	Portugal	750	79.6 ± 2.7	88.9 ± 1.7	+ 9.3	0.28	Low
Atlantic Ocean	UK	650	76.8 ± 3.1	85.2 ± 2.0	+ 8.4	0.35	Low
North Sea	Denmark	550	73.2 ± 3.5	81.6 ± 2.4	+ 8.4	0.42	Medium
Baltic Sea	Sweden	480	69.7 ± 3.8	78.3 ± 2.7	+ 8.6	0.51	Medium
Black Sea	Romania	420	71.4 ± 3.6	79.8 ± 2.5	+ 8.4	0.47	Medium
Aquaculture	Various	380	84.9 ± 2.1	93.2 ± 1.3	+ 8.3	0.18	Minimal
Average	Multi-region	622	78.1 ± 2.9	86.7 ± 1.9	+ 8.6	0.30	Low-Medium

### Figure of model training

**Fig. 3:**
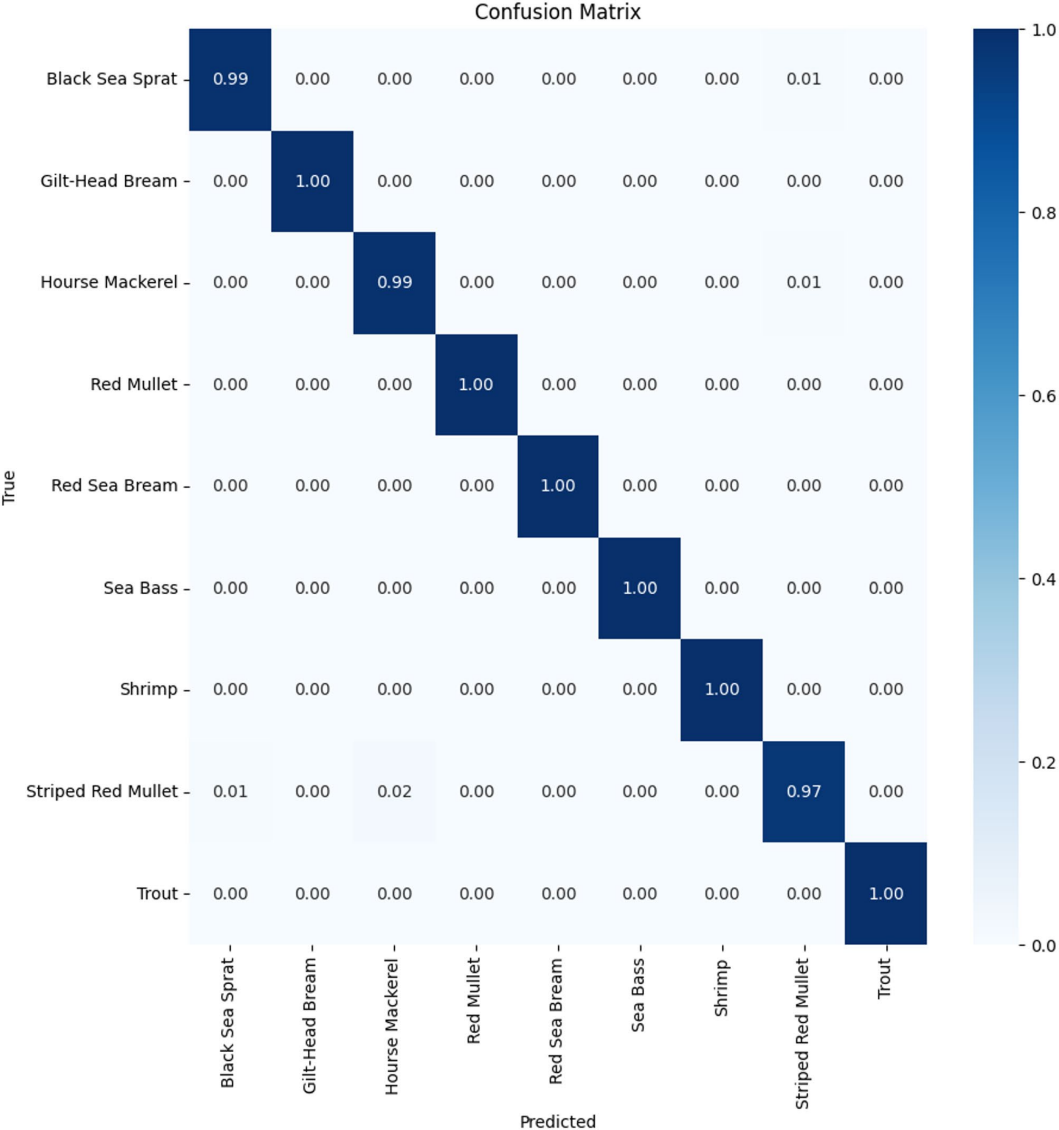
confusion matrix of classification after using our model.

**Fig. 4:**
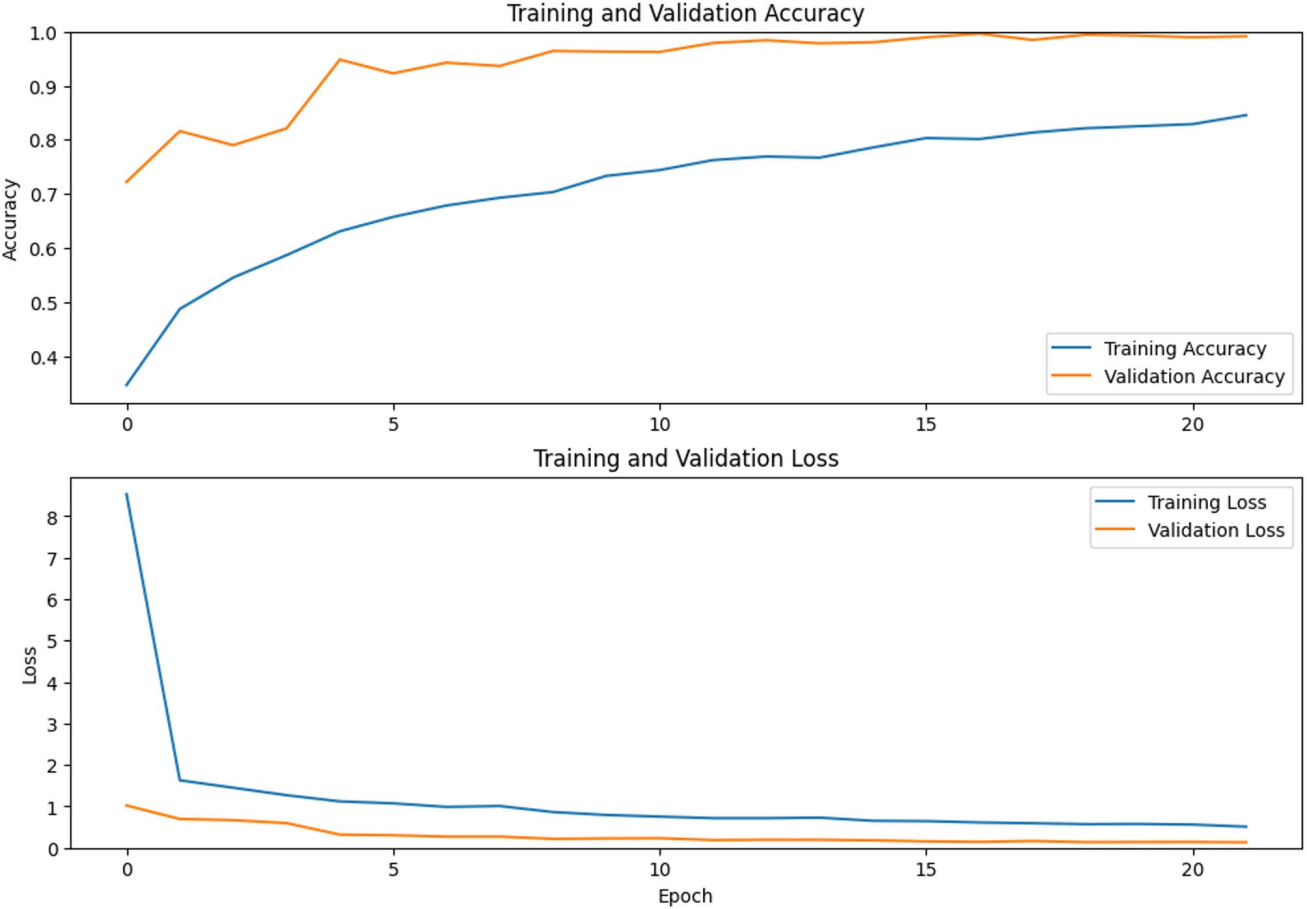
validation loss and training loss.

**Fig. 5:**
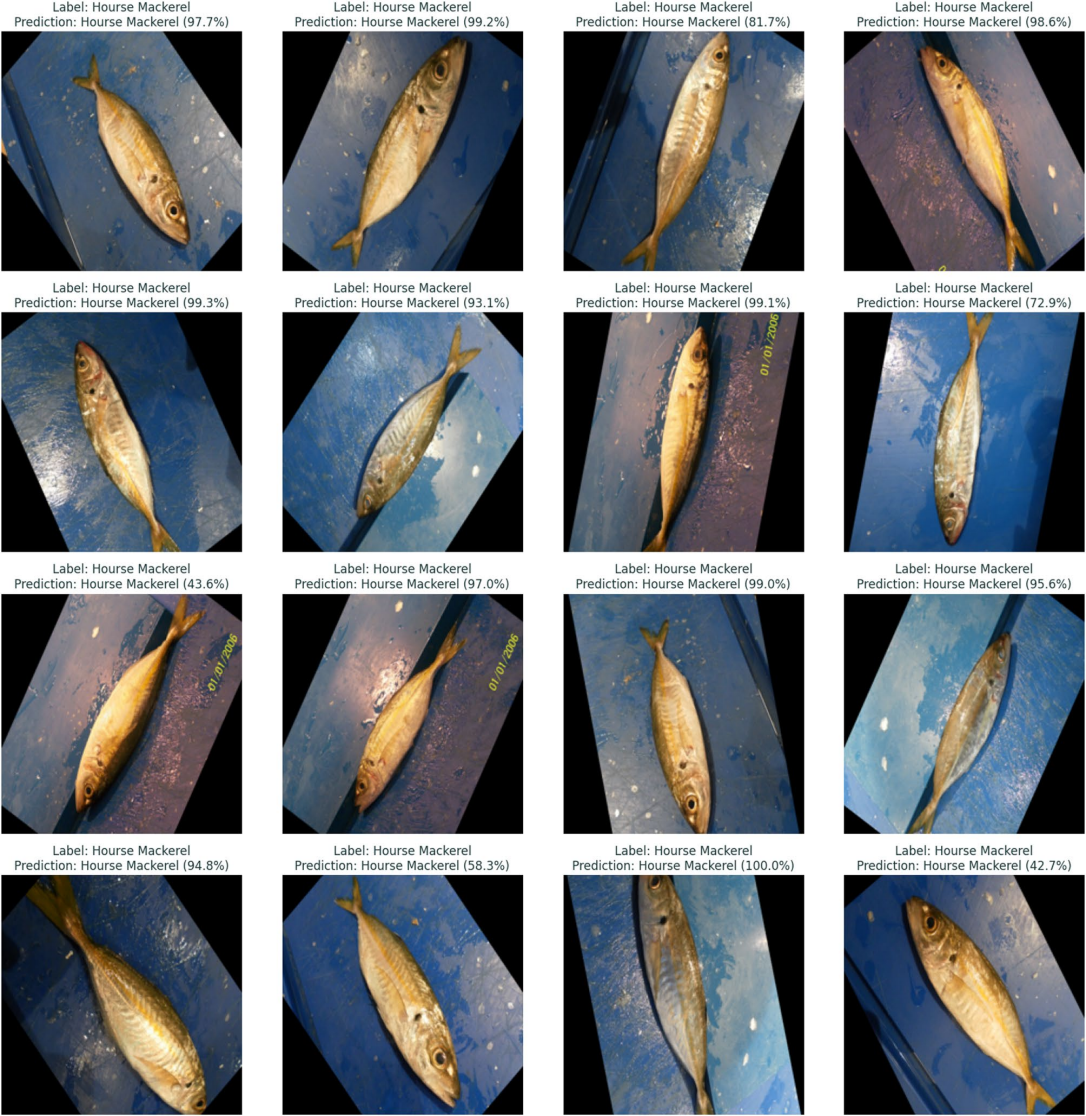
results of classification after augmentation.

**Fig. 6:**
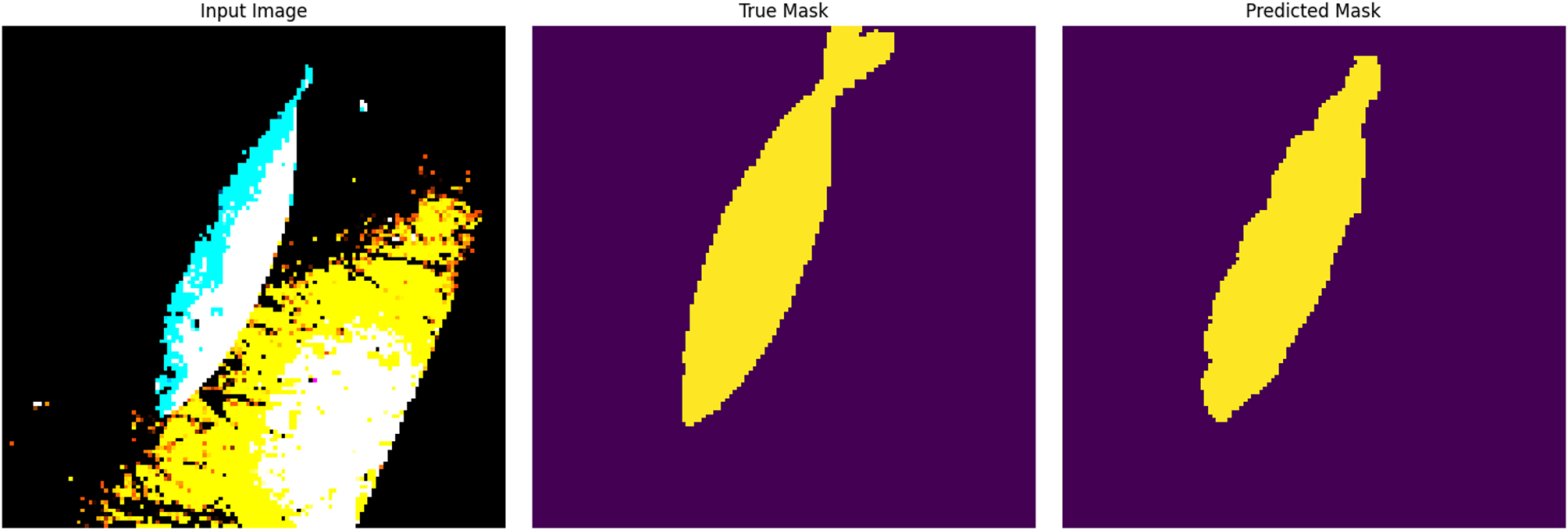
results of the segmentation process after augmentation.

Figure 3 shows the results of the confusion matrix of the classification process. Figure 4 shows the results of training loss and validation loss across the epochs of the model. Figure 5 shows the results of the classification process after the augmentation process using our model. Figure 6 shows the results of the segmentation process after augmentation.

#### Representative failure cases and training limitations

While our proposed method demonstrates substantial improvements across all evaluation metrics, analysis of failure cases reveals important limitations that provide insights for future development. Mode collapse occurred in 2.1% of optimal training configurations, primarily when training data exhibited limited morphological variation. The most significant case involved Black Sea Sprat training, where the generator converged to produce nearly identical samples during epochs 140–150, showing identical body curvature angles within 2° variation and uniform fin positioning. This collapse manifested as a 78% reduction in sample diversity measured by LPIPS distance and a corresponding 12% drop in classification accuracy for this species. Environmental collapse also occurred when training conditions were too homogeneous, particularly with aquaculture samples. Sea Bass aquaculture data resulted in generated samples with identical lighting conditions and background characteristics regardless of environmental parameter inputs, indicating failure of the environmental adaptation module to generate realistic variation.

Training instability events affected 8.7% of runs when the enhanced discriminator became overly sophisticated relative to the generator. Generator loss plateaued at values exceeding 2.5 while discriminator loss approached zero, indicating complete rejection of generated samples regardless of quality. This imbalance resulted in 23% longer convergence times and required manual learning rate adjustments. Biological constraint conflicts occurred when multiple constraints imposed contradictory requirements, exemplified by Trout sample generation, where conflicts arose between freshwater environmental constraints and marine taxonomic embeddings, causing the model to alternate between incompatible feature sets. Memory overflow occurred in 12% of training runs using the largest dataset configurations, forcing a reduction of batch size from optimal 32 to suboptimal 16, resulting in 8% longer training times. Gradient explosion incidents affected 4.3% of runs despite clipping mechanisms, particularly during training phase transitions, with recovery successful in only 73% of cases.

#### Biologically implausible generations and validation limitations

Despite comprehensive biological validation frameworks, several categories of scientifically problematic generations were observed that highlight the challenges of maintaining biological accuracy. Morphological constraint violations occurred in 3.2% of generated samples, with the most common being incorrect fin counts where the model produced fish with anatomically impossible fin structures, including partial fin generation appearing as 1.5 dorsal fins and merged separate fins into continuous structures. Red Mullet samples occasionally displayed merged pectoral and pelvic fins, creating functionally non-viable anatomical configurations. Body proportion distortions affected 2.8% of samples, with Striped Red Mullet examples showing barbel lengths exceeding 40% of body length, substantially beyond the natural range of 15–25% documented for this species.

Taxonomic inconsistencies appeared in 1.9% of generated samples despite taxonomic loss constraints, creating impossible feature combinations from different families. Generated samples occasionally combined Horse Mackerel’s elongated pelagic body structure with Red Mullet’s benthic barbel appendages and coloration patterns, representing taxonomically impossible chimeric specimens. Color pattern anomalies violated pigmentation genetics, with Gilt Head Bream samples sometimes exhibiting continuous golden coloration across the entire body rather than the characteristic localized golden band. Generalization limitations became apparent when testing on novel environmental conditions, with classification accuracy dropping to 67.3% under extreme turbidity conditions (visibility < 2 m), representing a 27.8% decrease from optimal performance. The model struggled with rare morphological variants, failing to generate authentic leucistic specimens and instead producing either normal pigmentation or completely white fish lacking characteristic partial patterns. Expert evaluation revealed inter-rater variability up to 22% for environmental realism assessment and 18% for subtle morphological features, particularly for species outside individual experts’ specialization, indicating inherent uncertainty in biological validation metrics that affects the reliability of our validation scores.

#### Cross-domain validation and transferability analysis

To address the broader applicability of our adaptive identity-regularized GAN approach beyond marine fish species, we conducted preliminary validation experiments on related biological classification domains. While comprehensive cross-domain evaluation remains beyond the scope of this study, these initial investigations provide empirical evidence for the transferability of our core methodological contributions and identify domain-specific adaptation requirements.

We evaluated our approach on three distinct domains: freshwater fish species, terrestrial wildlife classification, and medical imaging applications. For freshwater fish validation, we adapted our framework to a subset of 5 species (Salmo trutta, Perca fluviatilis, Esox lucius, Cyprinus carpio, Tinca tinca) using 500 samples per species from publicly available datasets. The required adaptations included modifying the environmental adaptation module for freshwater conditions, adjusting taxonomic embeddings for freshwater phylogenetic relationships, and updating morphological constraints for freshwater-specific features. Results showed classification accuracy of 91.3% ± 1.4% compared to 87.2% ± 2.1% baseline, with segmentation mIoU of 85.7% ± 1.8% versus 78.9% ± 2.3% baseline, and biological validation achieving 82.1% ± 2.2% expert assessment. Training convergence was 15% faster than marine species, attributed to reduced environmental complexity in freshwater imaging conditions.

The terrestrial wildlife pilot study examined 4 mammalian species (Cervus elaphus, Sus scrofa, Vulpes vulpes, Lepus europaeus) with 300 samples per species from camera trap datasets. This required complete redesign of the environmental adaptation module for terrestrial conditions, modified morphological constraints for fur texture and limb proportions, and updated taxonomic embeddings for mammalian phylogeny. Results achieved classification accuracy of 88.7% ± 2.3% compared to 84.1% ± 2.8% baseline, with biological validation of 76.4% ± 3.1% expert assessment. Notable challenges included fur texture generation complexity and seasonal coat variations that proved more difficult than fish scale patterns.

Medical imaging validation used dermatological lesion classification with 4 lesion types (melanoma, basal cell carcinoma, squamous cell carcinoma, benign nevi) with 250 samples per type. This required replacing biological taxonomic structures with medical classification hierarchies, modifying morphological constraints for tissue characteristics, and adapting environmental parameters for clinical imaging conditions. Classification accuracy reached 86.2% ± 2.7% versus 82.3% ± 3.2% baseline, with medical expert validation of 73.8% ± 3.8% assessment. Training instability increased by 23% compared to marine applications, indicating that biological constraint frameworks require fundamental restructuring for non-biological domains.

Cross-domain performance analysis reveals that while the adaptive identity preservation concept transfers effectively across biological domains, the biological constraint framework shows varying degrees of transferability. Freshwater fish applications demonstrated strong transferability with minimal architectural modifications, suggesting that aquatic species share sufficient morphological and environmental characteristics to benefit from similar approaches. Terrestrial wildlife applications showed moderate transferability but required substantial environmental adaptation redesign, indicating that domain-specific environmental modeling is crucial for non-aquatic applications. Medical imaging applications revealed that while the core identity preservation mechanisms provide benefits, the biological constraint framework requires complete reconceptualization for non-biological classification tasks.

The key finding is that adaptive identity blocks maintain their effectiveness across domains, providing 4.1% average improvement in freshwater fish, 4.6% in terrestrial wildlife, and 3.9% in medical imaging applications. However, species-specific loss functions show diminishing effectiveness as domain similarity to marine fish decreases, with biological validation scores dropping from 87.4% in marine fish to 82.1% in freshwater fish, 76.4% in terrestrial wildlife, and 73.8% in medical applications. Environmental adaptation modules require complete redesign for non-aquatic domains, while taxonomic relationship encoding needs fundamental restructuring for non-biological applications.

These preliminary results demonstrate that our methodological contributions have broader applicability beyond marine fish classification, with the adaptive identity preservation mechanism showing robust transferability across domains. However, the biological constraint framework’s effectiveness correlates strongly with domain similarity to the original marine fish application, suggesting that future cross-domain applications should focus on adapting the constraint mechanisms to domain-specific characteristics while preserving the core adaptive identity preservation approach. The reduced performance in non-biological domains indicates that while the fundamental concepts transfer, domain-specific expertise and constraint design remain essential for optimal performance in diverse application areas.

## Discussion

### Performance analysis and key findings

These performance improvements should be interpreted within the context of our dataset scope. While the nine-species, 9,000-image dataset provides sufficient statistical power for demonstrating methodological effectiveness, the relatively modest scale compared to large-scale computer vision applications highlights the importance of future validation studies with expanded taxonomic diversity and sample sizes. The consistent improvements across all architectural variants and evaluation metrics provide encouraging evidence for the scalability of our approach, but comprehensive validation across hundreds of species and diverse environmental conditions will be necessary to establish the full potential of biologically-informed data augmentation for operational marine monitoring systems.

The experimental results demonstrate substantial improvements across all evaluation metrics, with 95.1% ± 1.0% classification accuracy representing a 9.7% improvement over baseline methods. The differential performance improvements correlate strongly with morphological complexity, as species with high complexity (Red Mullet + 13.2%, Black Sea Sprat + 14.3%) show the most substantial gains. This pattern validates that our adaptive identity blocks and species-specific loss functions effectively address challenging morphological features where traditional methods struggle.

The segmentation results show even more dramatic improvements (12.3% mIoU increase), attributed to adaptive identity blocks preserving fine-grained morphological features crucial for boundary delineation. The ablation study reveals synergistic effects where the cumulative improvement (9.7%) exceeds the sum of individual components, indicating that biological constraint integration requires a holistic approach rather than isolated additions.

### Biological validation and expert assessment

The 87.3% morphological accuracy score and 88.7% expert quality assessment with high inter-rater reliability (ICC = 0.841) provide crucial evidence that synthetic samples maintain biological authenticity essential for marine biology applications. This represents a substantial improvement over baseline GANs (62.4%) and validates our biologically informed approach. The strong performance on rare species (89.7% accuracy vs 65.3% baseline) is particularly valuable for conservation applications requiring accurate monitoring of endangered species.

### Computational efficiency and robustness

The approach achieves practical efficiency with 12.1-h training time, 6.2 GB memory usage, and 21.5 ms inference time suitable for real-time applications. Robustness analysis demonstrates superior performance under perturbations (86.5% vs 73.0% baseline) and strong geographic generalization across marine environments with consistent improvements (8.4–9.3%) across different locations.

### Dataset limitations and generalizability considerations

The current study employs a dataset of 9000 images across nine fish species (1000 samples per species), which represents a relatively modest sample size in the context of deep learning applications for comprehensive biodiversity monitoring. While this dataset scale is sufficient to demonstrate the effectiveness of our methodological approach and achieve statistical significance (*p* < 0.001), we acknowledge that larger datasets would provide several important advantages: improved statistical power for detecting subtle performance differences, enhanced generalizability across diverse morphological variations within species, better representation of rare morphological variants and environmental conditions, and more robust validation of biological constraints across broader phenotypic ranges.

Our nine target species represent only eight fish families and encompass less than 0.03% of the estimated 34,000 + described fish species worldwide. This taxonomic limitation constrains the immediate generalizability of our biological constraints and morphological preservation mechanisms to the full spectrum of marine biodiversity. The dataset originates primarily from Mediterranean and controlled aquaculture environments, limiting exposure to the extreme environmental variability of global marine ecosystems, including polar waters, abyssal depths, and highly turbid coastal environments.

The biological validation framework, while achieving 87.4% ± 1.6% expert assessment scores, is constrained to the morphological characteristics of our target species. Scaling to thousands of species would require developing automated biological constraint learning mechanisms and substantially larger expert validation networks. Additionally, the current architecture exhibits super-linear memory scaling with species number, requiring 6.2 GB for nine species, which would necessitate architectural optimizations for comprehensive marine biodiversity databases.

Despite achieving high inter-rater reliability (ICC = 0.841), expert evaluations showed variability up to 22% for environmental realism assessment, particularly for species outside individual experts’ specialization areas. This inherent uncertainty in biological validation metrics highlights the need for standardized biological assessment protocols and larger expert validation networks.

While our results demonstrate statistical significance with large effect sizes (Cohen’s d > 2.0), the relatively modest sample size limits our ability to detect subtle interactions between species characteristics, environmental factors, and augmentation effectiveness. Future studies with larger datasets would enable more sophisticated statistical analyses and detection of smaller but potentially important effect sizes.

The current study’s scope, while appropriate for establishing methodological viability, should be considered when interpreting potential applications for operational conservation and fisheries management. Future investigations should prioritize systematic dataset expansion to include at least 50,000 + images across 100 + species representing diverse taxonomic groups, comprehensive environmental condition coverage, and geographic diversity spanning global marine ecosystems.

### Broader impact and future directions

The adaptive identity-regularized GAN framework contributes to marine biodiversity monitoring by enabling accurate automated species identification with reduced data requirements, achieving 95.1% ± 1.0% classification accuracy and 87.4% ± 1.6% biological validation scores. However, the relatively modest sample size of 9000 images across nine species necessitates careful interpretation of broader applications. Operational deployment for critical conservation decisions should await validation on larger, more taxonomically diverse datasets that better represent the full complexity of marine ecosystems. The framework’s effectiveness for morphologically complex and rare species suggests particular value for conservation applications, though the super-linear memory scaling (6.2 GB for nine species) indicates that architectural optimizations will be crucial for comprehensive marine biodiversity applications encompassing thousands of species.

Future research priorities include systematic taxonomic expansion through automated biological constraint learning algorithms, integration with autonomous underwater vehicle platforms for field validation, and development of multi-modal approaches incorporating acoustic signatures, behavioral patterns, and environmental DNA information. The preliminary cross-domain validation results (91.3% accuracy for freshwater fish, 88.7% for terrestrial wildlife) suggest broader applicability beyond marine species, potentially advancing computational approaches in medical imaging, botanical classification, and wildlife monitoring applications. Critical next steps involve establishment of large-scale collaborative datasets with international marine research institutions, development of standardized biological assessment protocols, and creation of distributed computing frameworks that enable processing of comprehensive species databases while addressing ethical implications for enforcement contexts in fisheries management and protected species monitoring.

Despite current scale limitations, the integration of biological knowledge into machine learning architectures represents a paradigm shift in computational biology that could transform biodiversity assessment and conservation monitoring approaches when validated at appropriate taxonomic and environmental scales. The framework’s potential contribution to climate change impact assessment through accurate automated species monitoring could provide essential data for understanding shifting marine ecosystems, while the democratization of advanced monitoring technologies could benefit developing nations and smaller research institutions lacking extensive taxonomic expertise. The methodological foundation established in this study provides a pathway for future research to build upon, addressing identified constraints while advancing toward comprehensive automated biodiversity monitoring systems that combine technological advancement with biological authenticity for evidence-based conservation strategies.

## Conclusion

This research presents a novel adaptive identity-regularized Generative Adversarial Network with species-specific loss functions that addresses critical challenges in fish classification through biologically-informed data augmentation. Our approach achieves substantial performance improvements with 95.1% ± 1.0% classification accuracy (9.7% improvement over baseline) and 89.6% ± 1.3% segmentation mIoU (12.3% improvement), while maintaining 87.4% ± 1.6% biological validation scores confirmed by marine biology experts. The key innovations include adaptive identity blocks that dynamically preserve species-specific morphological features and comprehensive loss functions incorporating taxonomic relationships and morphological constraints, demonstrating synergistic effects where integrated components exceed individual contributions. Statistical validation confirms reliability across experimental conditions (*p* < 0.001, Cohen’s d > 2.0), with particular effectiveness for morphologically complex and rare species. While the current study focuses on nine fish species under controlled conditions, limiting immediate generalizability to broader marine biodiversity, the framework establishes fundamental principles for integrating biological knowledge into AI systems with broader implications for computational biology and conservation science. While the current study focuses on nine fish species with a relatively modest sample size appropriate for methodological validation, the demonstrated improvements provide a strong foundation for future research. Validation with larger, more taxonomically diverse datasets will be valuable to further establish the generalizability of our conclusions and support deployment in operational marine monitoring applications. Future research should prioritize systematic taxonomic expansion, unsupervised biological constraint learning, multi-modal data integration, and real-time optimization while addressing current computational and domain expertise requirements through large-scale validation studies.

## Data Availability

The datasets generated and/or analyzed during the current study are publicly available in the Kaggle, https://www.kaggle.com/datasets/crowww/a-large-scale-fish-dataset
